# Non-centrosymmetric topological phase probed by non-linear Hall effect

**DOI:** 10.1093/nsr/nwad103

**Published:** 2023-04-24

**Authors:** Naizhou Wang, Jing-Yang You, Aifeng Wang, Xiaoyuan Zhou, Zhaowei Zhang, Shen Lai, Yuan-Ping Feng, Hsin Lin, Guoqing Chang, Wei-bo Gao

**Affiliations:** Division of Physics and Applied Physics, School of Physical and Mathematical Sciences, Nanyang Technological University, Singapore 637371; Department of Physics, National University of Singapore, Singapore 117551; Low Temperature Physics Laboratory, College of Physics and Center for Quantum Materials and Devices, Chongqing University, Chongqing 401331; Low Temperature Physics Laboratory, College of Physics and Center for Quantum Materials and Devices, Chongqing University, Chongqing 401331; Division of Physics and Applied Physics, School of Physical and Mathematical Sciences, Nanyang Technological University, Singapore 637371; Division of Physics and Applied Physics, School of Physical and Mathematical Sciences, Nanyang Technological University, Singapore 637371; Department of Physics, National University of Singapore, Singapore 117551; Centre for Advanced 2D Materials, National University of Singapore, Singapore 117546; Institute of Physics, Academia Sinica, Taipei 11529; Division of Physics and Applied Physics, School of Physical and Mathematical Sciences, Nanyang Technological University, Singapore 637371; Division of Physics and Applied Physics, School of Physical and Mathematical Sciences, Nanyang Technological University, Singapore 637371; The Photonics Institute and Centre for Disruptive Photonic Technologies, Nanyang Technological University, Singapore 637371

**Keywords:** non-linear Hall effect, topological insulator, ZrTe_5_

## Abstract

Non-centrosymmetric topological material has attracted intense attention due to its superior characteristics as compared with the centrosymmetric one, although probing the local quantum geometry in non-centrosymmetric topological material remains challenging. The non-linear Hall (NLH) effect provides an ideal tool to investigate the local quantum geometry. Here, we report a non-centrosymmetric topological phase in ZrTe_5_, probed by using the NLH effect. The angle-resolved and temperature-dependent NLH measurement reveals the inversion and ab-plane mirror symmetries breaking at <30 K, consistently with our theoretical calculation. Our findings identify a new non-centrosymmetric phase of ZrTe_5_ and provide a platform to probe and control local quantum geometry via crystal symmetries.

## Teaser

A non-centrosymmetric topological phase is discovered in ZrTe_5_, by a highly sensitive non-linear Hall effect to probe the symmetry breaking.

## INTRODUCTION

Since the discovery of the quantum Hall effect, topological states among the condensed matter materials have generated widespread interest owing to their scientific significance and potential for next-generation quantum devices [1–8]. According to whether the material has inversion symmetry, topological materials can be divided into centrosymmetric and non-centrosymmetric types [9]. Recently, the non-centrosymmetric topological material attracted intense interest due to its non-trivial phenomena as well as its potential in topological electronic devices. Due to the broken inversion symmetry, many exotic properties are predicted to emerge in non-centrosymmetric topological materials, such as topological p–n junctions [10], topological magneto-electric effects [11] and surface-dependent topological electronic states [12]. Furthermore, by driving a non-centrosymmetric topological insulator into a superconducting phase, the large upper critical field beyond the Pauli limit as well as topological superconductivity with Majorana edge channels can be realized [13,14]. All these non-trivial phenomena make the non-centrosymmetric topological material an ideal platform for topological electronics devices and quantum information processing.

Compared with a centrosymmetric topological material, the global topological index does not change in the non-centrosymmetric counterpart [15,16]. However, due to the difference in local geometric properties of the quantum wave function, a series of non-linear electromagnetic responses, e.g. non-linear Hall (NLH) effects and non-linear photocurrents, will emerge in a non-centrosymmetric topological insulator [17–23]. The NLH effect is intrinsically emerging from the Berry curvature dipole (BCD) moment of materials. Different from the linear Hall effect, the NLH effect does not need broken time-reversal symmetry but requires broken inversion symmetry [18]. Considering the high sensitivity of electrical signals, utilizing the NLH effect to probe the symmetry breaking will be highly feasible and effective. To date, however, the observation of the NLH effect is limited to the known non-centrosymmetric materials with finite BCD [17–19,22,23]. In this work, we predict and discover a new non-centrosymmetric structure ZrTe_5_ with the space group of Pna*2_1_*, which is obtained by slightly translating the ZrTe_3_ chain along the *x*-axis and displacement of Te atoms. The inversion symmetry breaking is observed to emerge at <30 K, leading to the NLH effect. The angle-resolved NLH measurement confirms the *xy*-plane mirror symmetry breaking at <30 K, consistently with our theoretical prediction. The symmetry breaking is further confirmed by the non-reciprocal transport measurement.

## RESULTS AND DISCUSSION

### The non-centrosymmetric topological phase in ZrTe_5_

**Figure 1.**
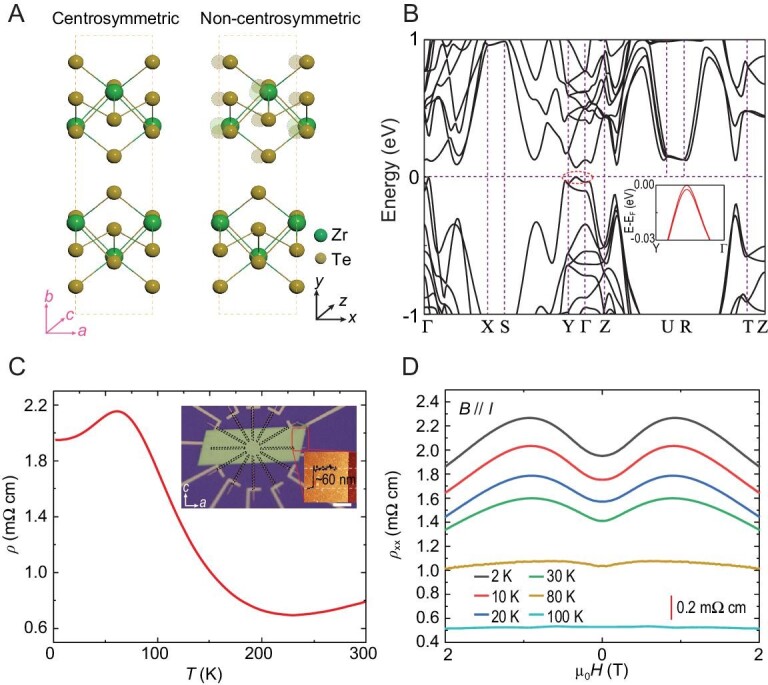
The non-centrosymmetric topological phase in ZrTe_5_. (A) The comparison of crystal structures between centrosymmetric and non-centrosymmetric ZrTe_5_. For the non-centrosymmetric ZrTe_5_, the adjacent layer is shifted along the *x*-axis for a small distance. In order to clearly describe the results, we set a cartesian *x, y* and *z* where *x, y* and *z* correspond to the three principal crystal axes *a, b* and *c*, respectively. (B) The calculated band structure of non-centrosymmetric ZrTe_5_. A valance band splitting could be observed as shown in the inset. (C) The temperature-dependent resistivity of the circular disc ZrTe_5_ device. The current is applied along the *x*-axis. The inset shows the optical photo of the device with AFM images. The thickness of the sample is determined to be 60 nm. Scale bar, 10 μm. (D) The magnetic-field-dependent resistivity of the ZrTe_5_ device, with the magnetic field applied along the current direction. A negative magnetoresistance could be observed at <100 K. To make the plots clearer, a vertically shift of 0.2 mΩ cm is added.

The transition metal pentatelluride ZrTe_5_ has recently attracted much attention because of its non-trivial topological properties [24–29]. It is predicted that monolayer ZrTe_5_ is a quantum spin Hall insulator with a large bandgap [24]. ZrTe_5_ is considered to have an orthorhombic layered structure with the space group of Cmcm (D^17^_2h_) [24]. As shown in Fig. 1A, the crystal structure consists of alternate stacking of 2D layers along the *y*-axis. Here, in order to clearly describe the results, we define cartesian *x, y* and *z* that correspond to the three principal crystal axes *a, b* and *c*, respectively. In each 2D layer, a ZrTe_6_ triangular prism forms a 1D ZrTe_3_ chain along the *x*-axis. The additional Te ions connect them and consequently form zigzag chains along the *x*-axis, making the crystal tend to grow along the *x*-axis. However, by translating one of the ZrTe_5_ layers stacked in the *y*-axis a small amount along the *x*-axis relative to the other layer accompanied by the displacement of Te atoms, we find an energetically more preferred non-centrosymmetric ZrTe_5_ phase in the space group of Pna2_1_. Figure 1A shows the structural comparison between the previously reported centrosymmetric ZrTe_5_ and our predicted non-centrosymmetric one [24]. The average energy of each atom in the non-centrosymmetric one is ∼2.7 meV lower than that in the centrosymmetric one. The energy difference between the non-centrosymmetric and centrosymmetric phases indicates the structural phase transition may occur at ∼31 K (Δ*E*/*k*_B_). The detailed lattice information of centrosymmetric and non-centrosymmetric ZrTe_5_ is listed in Supplementary Tables S1 and S2, respectively. The band structure of non-centrosymmetric ZrTe_5_ was calculated as shown in Fig. 1B. We found band splitting (see inset of Fig. 1B), which is expected in a system of broken central inversion symmetry.

In our experiment, high-quality ZrTe_5_ samples are studied. The circular disc devices with 12 electrodes are used (inset of Fig. 1C). Since the ZrTe_5_ crystal tends to grow along the *x*-axis and the exfoliation normally will produce rectangular flakes, we can easily determine the crystal axis and align it to the electrode direction. Figure 1C shows the temperature-dependent resistivity (*RT*) curve of the ZrTe_5_ (∼60 nm thick) sample when the current is applied along the *x*-axis. A resistivity peak is observed at ∼60 K, which is attributed to slight doping in the thin-flake samples (see Supplementary Section S4) [30]. Negative longitudinal magnetoresistance (LMR) under parallel magnetic and electric fields is usually considered as the signature of topological quasiparticles, such as bulk Weyl/Dirac fermions and surface Dirac cones of topological insulators [15,27,31–34]. Figure 1D shows the magnetic-field-dependent resistance for ZrTe_5_ when the magnetic field is aligned with both the current direction and the *x*-axis of the crystal. A negative LMR emerges below ∼100 K. The negative LMR is highly sensitive to the angle between the current direction and the magnetic field direction, which is thought to be related to the chiral magnetic effect [27,34] (see SupplementaryFig. S16). Therefore, we attribute the negative magnetoresistance in ZrTe_5_ to be associated with a topological non-trivial order. Our first-principles calculations also suggest that ZrTe_5_ is a topological insulator (TI) (see Supplementary Fig. S3).

### NLH effect driven by non-centrosymmetric crystal structure

**Figure 2.**
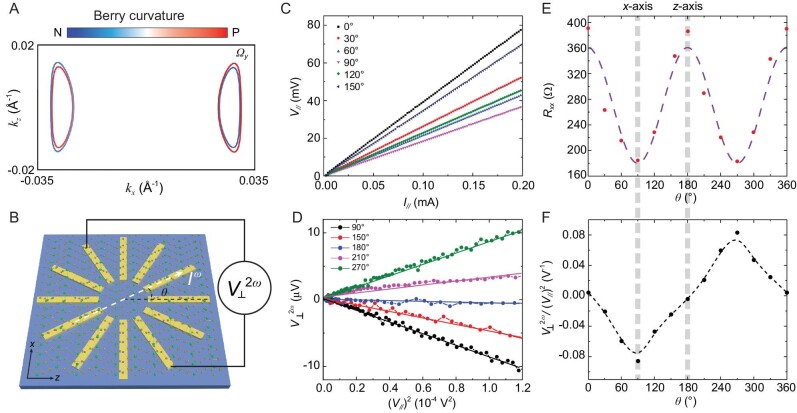
The NLH effect in ZrTe_5_. (A) The Fermi surface and Berry curvature distribution calculated based on the non-centrosymmetric ZrTe_5_. The Fermi level is fixed at –0.01 eV relative to the valence band maximum, which is determined from the SdHO measurement (see Supplementary Section S5). (B) The schematic view of the circular disc ZrTe_5_ device. The injected current is applied at an angle *θ* deviating from the *z*-axis and the transverse voltage is measured. (C) The first-harmonic *I–V* curves for the circular disc ZrTe_5_ device, with injected current along different directions. (D) The linear dependence of the second-harmonic transverse voltage $V_ \bot ^{2\omega }$ on the square of the first-harmonic longitudinal voltage ${V}_\parallel $, with injected current along different directions. The round symbols are the experimental data and the dashed lines are the linear fitting results. (E) The first-harmonic longitudinal resistance as a function of *θ* in the circular disc ZrTe_5_ device*. θ* is the injected current angle measured from the *z*-axis. The solid circle is the experimental data and the dashed line is the fitting result from Equation (2). (F) The second-harmonic Hall response as a function of *θ* in the circular disc ZrTe_5_ device. The solid circles are the experimental data and the dashed line is the fitting result from Equation (3). The error bars are smaller than the symbol.

Next, we investigate the local change in the geometric properties for ZrTe_5_ in momentum space. To expose the central inversion symmetry breaking in ZrTe_5_, we employ the NLH measurement to confirm the redistributed quantum wave function: the BCD. The NLH effect has shown its potential to probe crystal symmetry with high sensitivity and accuracy in a 2D system such as a few layers of WTe_2_ and twisted WSe_2_ [17–19,22,23]. However, unlike the NLH effect in 2D systems, we consider the 3D nature of the NLH effect in non-centrosymmetric ZrTe_5_. In a 3D system, the NLH current related to the BCD can be represented as ${\boldsymbol{J}}_{\boldsymbol{a}}^{2{\boldsymbol{\omega }}} = {{\boldsymbol{\chi }}}_{{\boldsymbol{abc}}}\ {\boldsymbol{E}}_{\boldsymbol{b}}^{\boldsymbol{\omega }}{\boldsymbol{E}}_{\boldsymbol{c}}^{\boldsymbol{\omega }}{\mathrm{\ }}$(Equation 1), where ${{\boldsymbol{\chi }}}_{{\boldsymbol{abc}}}$ is the non-linear tensor and the *E* is the external electric field [18]. Based on the symmetry analysis of non-centrosymmetric ZrTe_5_, the only left non-linear tensors are ${{\boldsymbol{\chi }}}_{{\boldsymbol{caa}}}$ and ${{\boldsymbol{\chi }}}_{{\boldsymbol{aac}}}$. Furthermore, the non-linear tensor ${{\boldsymbol{\chi }}}_{{\boldsymbol{abc}}}$ is related to the BCD ${{\boldsymbol{D}}}_{{\boldsymbol{ab}}}$. Thus, this NLH current ${\boldsymbol{J}}_{\boldsymbol{c}}^{{\boldsymbol{NLHE}}}{\mathrm{\ }}$is proportional to ${{\boldsymbol{D}}}_{{\boldsymbol{ab}}}$. Figure 2A shows the distribution of the Berry curvature contour for certain energy$( {{{\boldsymbol{\Omega }}}_{\boldsymbol{y}}} )$ in k-space for the non-centrosymmetric ZrTe_5_ crystal structure. Our calculation shows anisotropic Berry curvature contours. As a consequence, the BCD emerges. Hence, when the external electric field is applied along the *x*-axis, the NLH response can be measured along the *z*-axis.

To perform the non-linear transport measurement on the device, an AC current is applied at a fixed frequency (17.777 Hz) along a selected direction of the device. The longitudinal and transverse voltages at both the fundamental and second-harmonic frequencies are measured simultaneously. The angle-dependent measurement is carried out using disk geometry under a zero magnetic field at 2 K. The current is injected along one of the 12 electrodes with angle *θ* specified as the direction deviated from the *z*-axis, as shown in Fig. 2B. To determine the crystal axis as well as the in-plane anisotropy, we analysed the first-harmonic longitudinal voltage with different current injection directions. As shown in Fig. 2C, the voltage shows good linear dependence on the injected current at all angles, suggesting excellent ohms contact in each direction. The longitudinal resistance with different angles *θ, R*_//_(=*V*_//_/*I*_//_) is shown in Fig. 2E. The angle-dependent longitudinal resistance(*R*_//_(*θ*)) shows a 2-fold angular dependence, which is consistent with the ZrTe_5_ symmetry and a previous report [35]. By fitting the curve using the formula *R*_//_(*θ*) = *R_a_*sin^2^*θ + R_c_*cos^2^*θ* (*R_a_* and *R_c_* denoted as the resistance along the *x*-axis and *z*-axis, respectively) (Equation 2), the in-plane resistance anisotropy coefficient *r* (*r* = *R_x_*/*R_z_*) is obtained as ∼0.5. We then focus on the second-harmonic part. Indeed, consistently with our predictions, we find that the second-harmonic transverse voltage at the *z*-axis is non-zero when the current is along the *x*-axis and obeys a linear dependence with the square of the current, equivalently, *V*_//_, as shown in Fig. 2D. Besides, with an injected current applied along a different direction, the second-harmonic voltage varies. When reversing the current direction along the *x*-axis (90° and 270°), the second-harmonic voltage changes its sign, excluding the contribution from sample heating. We have also excluded other possible extrinsic effects to cause the second-harmonic response such as capacitive coupling, contact junctions, flake shape and thermoelectric effects (see Supplementary Section S7). The slope of *V*_⊥_^2ω^ vs (*V*_//_)^2^ as the function of angle *θ* is summarized in Fig. 2F. Unlike the first-harmonic response, which shows a 2-fold angular dependence (Fig. 2E), the second-order response only shows a 1-fold dependence. The maximum second-harmonic response is achieved when the current is injected along the *x*-axis and vanishes when the current is applied along the *z*-axis.

With the non-centrosymmetric ZrTe_5_ structure, we further fit the angle-resolved non-linear response through the second-order non-linear susceptibilities as follows (see ‘Materials and methods’ for detailed derivation):


(3) \begin{eqnarray*}
{\mathrm{\ }}\frac{{V_ \bot ^{2\omega }}}{{{{\left( {{V}_\parallel } \right)}}^2}} = {\rho }_c\ \sin\theta \cdot \frac{{ - 2{{\cos }}^2\theta {d}_{15}{\gamma }^2 + {d}_{31}{\gamma }^2{{\sin }}^2\theta }}{{{{\left( {\cos^2\theta + \gamma \sin^2\theta } \right)}}^2}},
\end{eqnarray*}

where the *d_ij_* is the non-vanishing element of the second-order non-linear susceptibility tensor $\chi _{ijk}^{( 2 )}$ for the non-centrosymmetric ZrTe_5_ [36]. Due to the global factor sin*θ*, it can be inferred that the NLH response is maximal when the driving current is applied perpendicular to the polar *z*-axis. The dashed line in Fig. 2D shows the fitting result with the above equation, which perfectly captures the experimental data. The observed experimental results prove the inversion symmetry breaking in ZrTe_5_ and matches our theoretical prediction.

### Temperature-driven phase transition

**Figure 3.**
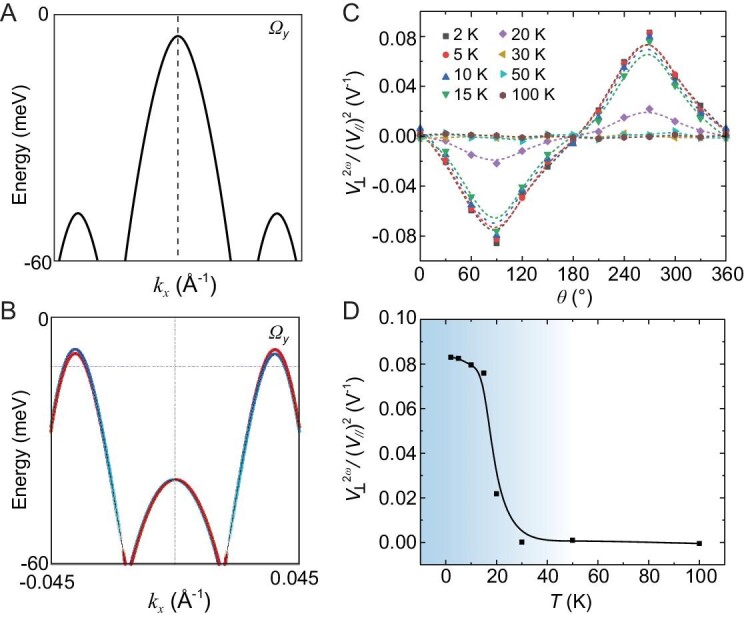
The temperature-driven non-centrosymmetric phase transition in ZrTe_5_. (A) The distribution of Berry curvature *Ω_y_* for centrosymmetric ZrTe_5_. The spin-up and spin-down states with opposite Berry curvature overlap with each other due to the spin degeneracy. (B) The distribution of Berry curvature *Ω_y_* for non-centrosymmetric ZrTe_5_. Spin-degeneracy is lifted due to the inversion breaking. Thus, a non-zero BCD emerges in the non-centrosymmetric structure. (C) The second-harmonic Hall response as a function of *θ* at different temperatures. The solid symbols are the experimental data and the dashed lines are the fitting result from Equation (3). The second-harmonic Hall response only emerges when the temperature is <30 K. (D) The temperature-dependent second-harmonic Hall response with injected current along the *x*-axis. The second-harmonic Hall response suddenly drops at >30 K and remains at nearly zero up to 100 K.

We further demonstrate the temperature-driven phase transition for ZrTe_5._ Figure 3A and B shows the distribution of Berry curvature for centrosymmetric and non-centrosymmetric ZrTe_5_ structures, respectively. The Fermi energy is determined based on the Shubnikov–de Haas (SdH) oscillation results (see Supplementary Section S5). Inversion symmetry breaking lifts the spin-degeneracy originally preserved in the centrosymmetric phase (Fig. 3A) and creates a Berry curvature pseudovector in momentum space (Fig. 3B). Our first-principles calculations indicate that the structural phase transition between the non-centrosymmetric and centrosymmetric phases occurs at ∼31 K. The NLH effect measurement can probe the redistribution of the quantum wave function. To confirm this, we perform temperature-dependent measurements. Figure 3C shows the angle-resolved second-harmonic Hall voltage at different temperatures, with a dashed line showing the fitting result with Equation (3). The second-harmonic Hall response gradually decreases with increasing temperature. We further plot the temperature-dependent second-harmonic Hall response with injected current along the *x*-axis in Fig. 2D. With the increase in temperature, the second-harmonic Hall voltage gradually decreases; it suddenly vanishes at >30 K and remains at zero up to 100 K. This phenomenon could be reproduced in several devices (see Supplementary Section S6). The second-harmonic Hall response usually follows the scaling behavior with the conductivity, which can be expressed as:


(4) \begin{eqnarray*}
\frac{{V_ \bot ^{2\omega }}}{{{{\left( {{V}_\parallel } \right)}}^2}} \propto \xi {\sigma }^2 + \eta ,
\end{eqnarray*}

where *σ* is the conductivity, and *ξ* and *η* are the constants. For ZrTe_5_, its conductivity remains nearly unchanged in the temperature range of 2–30 K, while the second-harmonic Hall response shows a tremendous change and is different from the description of Equation (4). The agreement between experimental data and first-principles calculations provides strong evidence for our experimental observation of a temperature-driven phase transition from centrosymmetric to non-centrosymmetric in ZrTe_5_ at <30 K.

**Figure 4.**
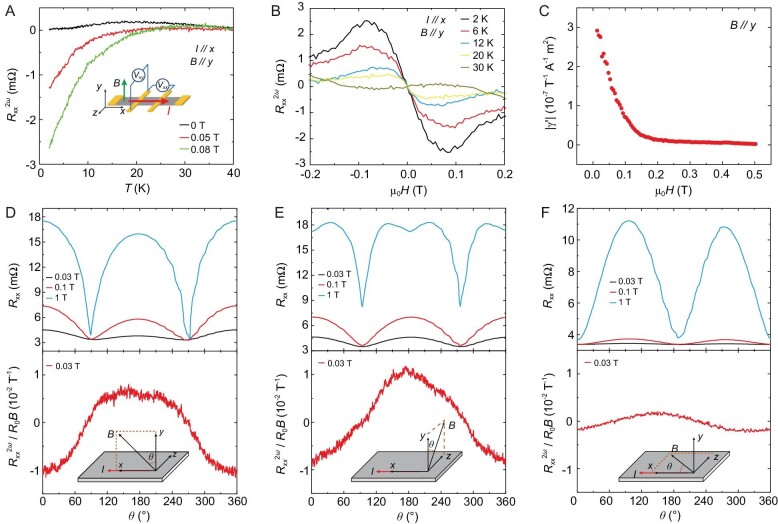
The non-reciprocal transport in ZrTe_5_. (A) The temperature-dependent second-harmonic longitudinal resistance of the bulk ZrTe_5_ sample at different magnetic fields. The inset shows the schematic view of the Hall bar configuration, where the current is applied along the *x*-axis and the magnetic field along the *y*-axis. (B) The magnetic-field-dependent second-harmonic longitudinal resistance of the bulk ZrTe_5_ sample at various temperatures. The second-harmonic longitudinal resistance gradually decreases with the increase in temperature and vanishes at >30 K. (C) The magnetochiral anisotropy coefficient of ZrTe_5_ as a function of the magnetic field. The current is applied along the *x*-axis and the magnetic field along the *y*-axis. (D)–(F) Magnetic-field-orientation-dependent resistance and normalized non-reciprocal response $R_{xx}^{2\omega }/{R}_0B$ at different magnetic fields, with the magnetic field rotating in the *xy, yz* and *xz* planes, respectively. All data are measured at *T* = 2 K. The rotation plane and the definition of the rotated angle *θ* are shown in each panel.

In order to exclude the possibility that the phenomenon observed in thin-flake ZrTe_5_ is due to the defect or degradation of the sample during fabrication or even an interfacial interaction, we perform systematic non-reciprocal transport measurement on a bulk ZrTe_5_ sample. For an inversion symmetry broken system, when under a magnetic field, a non-reciprocal transport effect could be observed [37]. Depending on the specific crystal symmetry, the non-reciprocal resistance could be divided into two types: the chiral structure type with $R\ = {R}_0\ [ {1 + \gamma ( {{\boldsymbol{B}} \cdot {\boldsymbol{I}}} )} ]$, where ${R}_0$ is the reciprocal resistance and $\gamma $ is a coefficient; and the polar structure with $R\ = {R}_0\ [ {1 + \gamma ( {{\boldsymbol{P}} \times {\boldsymbol{B}}} ) \cdot {\boldsymbol{I}}} ]$, where ***P*** is the unit vector that shows the direction of polarization in the structure. The coefficient $\gamma $($\gamma = [ {( {\frac{R}{{{R}_0}}} ) - 1} ]/( {| B | \cdot | I |} )$), for ${\boldsymbol{B}}\parallel {\boldsymbol{I}}$ with the chiral structure and $( {{\boldsymbol{P}} \times {\boldsymbol{B}}} )\parallel {\boldsymbol{I}}$ for the polar structure, could be used to evaluate the magnetochiral anisotropy in the material [37]. Figure 4A shows the second-harmonic longitudinal resistance $R_{xx}^{2\omega }$ of the bulk ZrTe_5_ sample under different magnetic fields, with the current along the *x*-axis and the magnetic field along the *y*-axis. The second-harmonic resistance $R_{xx}^{2\omega }$ only emerges at <30 K and increases with the increase in the magnetic field, which confirms the inversion symmetry breaking in ZrTe_5_. Moreover, the second-harmonic longitudinal resistance $R_{xx}^{2\omega }$ shows quadratical dependence on the applied current and antisymmetry with the magnetic field, which is in accordance with the non-reciprocal response of the polar structure with $R\ = {R}_0 [ {1 + \gamma ( {{\boldsymbol{P}} \times {\boldsymbol{B}}} ) \cdot {\boldsymbol{I}}} ]$ (see Supplementary Section S8). Figure 4B shows the magnetic-field-dependent second-harmonic longitudinal resistance $R_{xx}^{2\omega }$ under different temperatures. With μ_0_*H* < 0.08 T, the $R_{xx}^{2\omega }$ increases with the increase in the magnetic field. As the temperature increases, the $R_{xx}^{2\omega }$ gradually decreases and finally vanishes at 30 K, which suggests that the inversion symmetry breaking happened at <30 K. Our finding in bulk ZrTe_5_ samples is totally consistent with what is observed in the thin-flakes sample despite different methods, which proves that the centrosymmetric-to-non-centrosymmetric phase transition in ZrTe_5_ is intrinsic. Moreover, a giant magnetochiral anisotropy coefficient, $| {\gamma ^{\prime}} | = | {2{A}_ \bot {R}_{2\omega }/( {{R}_0B{I}_0} )} |\ $, with the magnetic field along the *y*-axis is observed at low fields. We noticed a recent report that also observed a giant magnetochiral anisotropy coefficient as well as the emergence of $R_{xx}^{2\omega }$ at <20 K corresponding to the symmetry breaking, which is consistent with our result [38]. Figure 4D–F shows the measurement of ${R}_{xx}$ and $R_{xx}^{2\omega }$ under different magnetic fields, with varying different rotating planes along the *xy, yz* and *xz* planes, respectively. For ${R}_{xx}$, the resistance shows a 2-fold dependency on the angle *θ* within the *xy, yz* and *xz* planes. As depicted from the equation that $R\ = {R}_0 [ {1 + \gamma ( {{\boldsymbol{P}} \times {\boldsymbol{B}}} ) \cdot {\boldsymbol{I}}} ]$, we can determine the polar axis by measuring $R_{xx}^{2\omega }$ with the magnetic field rotating along different crystalline planes. In order to prevent the influence of a reciprocal response, $R_{xx}^{2\omega }$ is normalized by ${R}_0B$. For the *xy*-plane and *yz*-plane, $R_{xx}^{2\omega }$ follows a cos*θ* dependence, where *θ* is the angle by which the magnetic field deviates from the *y*-axis. In contrast, $R_{xx}^{2\omega }$ remains at almost zero with a rotating magnetic field within the *xz*-plane. Since the current is along the *x*-axis, it suggests that the polar axis ***P*** is along the *z*-axis, in accordance with the results from NLH measurement and first-principles calculation.

## CONCLUSION

In summary, our work highlights a phase transition from a centrosymmetry to a non-centrosymmetry structure at 30 K in ZrTe_5_. The symmetry breaking leads to local quantum geometry redistribution. The phase transition is probed by using a temperature-dependent, angle-resolved 3D NLH effect, as well as non-reciprocal transport measurement. Moving forward, the observation of NLH in ZrTe_5_ also provides many possibilities. First, based on our theoretical calculation (see Supplementary Section S15), the BCD and NLH response of ZrTe_5_ could be dramatically tuned by the charge carrier density. Moreover, applying strain could switch the ZrTe_5_ from strong TI to weak TI, which will lead to the change in the BCD and the NLH effect [39]. Second, our results provide a valuable tool to identify and control the local quantum geometry via crystal symmetries, which could be extended to a broad range of topological materials. Finally, our finding paves the way to realizing more interesting applications such as signal rectification or frequency doubling, topological p–n junctions [10] and topological magneto-electric effects [11].

## MATERIALS AND METHODS

### Sample fabrication

High-quality ZrTe5 samples are synthesized by using a Te-flux method [27,28]. High-purity zirconium and tellurium are mixed with the atomic ratio of Zr : Te = 1 : 50, then loaded into a quartz ampoule and sealed under vacuum. The ampoule is heated to 550°C and kept for 10 h to homogenize the melt. It is cooled down to 460°C in 100 h. ZrTe_5_ crystals are isolated from the Te flux by centrifuging at 460°C. For the thin-flake samples, two types of devices are employed, including the standard Hall bar set-up as well as a circular disc set-up with 12 electrodes. The ZrTe_5_ thin flakes are mechanically exfoliated from the bulk crystal onto polydimethylsiloxane and then released onto SiO2/Si substrates with pre-patterned Cr/Au electrodes, followed by stacking hBN or spinning a thin layer of poly(methyl methacrylate) PMMA to prevent possible degradation. The ZrTe_5_ thin flakes are identified by using optical microscopy and the thickness is measured by using atomic force microscopy (AFM). The crystal orientation is determined by flake. For the bulk ZrTe_5_ sample, the needle-like samples with typical dimensions of 2 × 0.2 × 0.1 mm are used. To achieve good electrical contact, 100 nm of Au is deposited to form a Hall bar set-up by using a mask.

### Transport measurement

Electrical transport measurements are carried out in a close-cycled cryostat (Cryomagnetic) with a base temperature of ∼2 K and magnetic field of ≤7 T. Some experiments are carried out in a physical property measurement system (PPMS, Quantum Design) with a magnetic field of ≤14 T. Both first- and second-harmonic signals are collected by using standard lock-in techniques (Zurich MFLI) with excitation frequencies ranging from 10 to 200 Hz. The data shown in the manuscript are collected at a low frequency (17.777 Hz). During the transport measurements, the phase of the first- and second-harmonic signals are confirmed to be ∼0 and ∼90, respectively.

### Symmetry analysis on the NLH effect of ZrTe_5_

The BCD, *D_bd_*, can be expressed as [18,40]:


(5) \begin{eqnarray*}
{\mathrm{\ }}{D}_{bd} = \sum_n\ \mathop \int \nolimits_k v_b^n\Omega _d^n\left( k \right)\left( { - \frac{{\partial {f}_0\!\left( k \right)}}{{\partial {E}^n\!\left( k \right)}}} \right),
\end{eqnarray*}

where ${E}^n\ {\mathrm{and}}\ v_b^n$ are the band energy and Fermi velocity of the *n*-th band and $\Omega _d^n$ is the Berry curvature pseudovector defined by:


(6) \begin{eqnarray*}
\Omega _d^n = \ - 2{\hbar }^2{\epsilon }_{abd}\mathop \sum \limits_{m \ne n} Im\frac{{\left \langle {nk\left| {\frac{{\partial H}}{{\partial {k}_a}}} \right|mk} \right \rangle \ \left \langle {mk\left| {\frac{{\partial H}}{{\partial {k}_b}}} \right|nk} \right\rangle }}{{{{\left( {{E}_{nk} - {E}_{mk}} \right)}}^2 + i\eta }}.
\end{eqnarray*}

In order to have the BCD in a time-reversal symmetry preserved system, the inversion symmetry must be broken, since $\Omega $ and $v_b^n$ are even and odd functions under the inversion symmetry, respectively. As a result, the total integral of the BCD in the whole Brillouin zone is zero if both time-reversal and inversion symmetries are preserved.

The original ZrTe_5_ is the centrosymmetric space group Cmcm (No. 63). Here, we consider an intermediate structure of ZrTe_5_ that breaks inversion symmetry and its point group is C_2v_ with the symmetry operators of (*I, C_2z_, M_x_, M*). For *M_x_*, the Fermi velocity *v_x_* is an odd function, while *v_y_* and *v_z_* are even functions. Because the Berry curvature $\Omega $ is a pseudovector, ${\Omega }_x,{\Omega }_y\ {\mathrm{and}}\ {\Omega }_z$ under *M_x_* operation are even, odd and odd functions, respectively. Similar analysis can be used for *M_y_*. The symmetry consideration of the mirror symmetry of the Fermi velocity and Berry curvature are shown in Table 1.

**Table 1.** The properties of Fermi velocity and Berry curvature under mirror symmetry operators.

Fermi velocity/Berry curvature	*v_x_*	*v_y_*	*v_z_*	${\Omega }_x$	${\Omega }_y$	${\Omega }_z$
Property of *M_x_*	Odd	Even	Even	Even	Odd	Odd
Property of *M_y_*	Even	Odd	Even	Odd	Even	Odd

Having illustrated the symmetry effects on the Fermi velocity and Berry curvature, the behavior of the BCD can be easily understood. For example, since *v_x_* is an odd function while ${\Omega }_x$ is an even function under *M_x_* operation, *D_xx_* is constrained to be zero because *D_xx_* ∼ $\mathop \smallint \nolimits_k {v}_x\Omega _x^n( k )$, and the integral of an odd function and an even function in the whole Brillouin zone is zero.

Therefore, only *D_xy_, D_yx_, D_xz_* and *D_zx_* are permitted under *M_x_*; other components of the BCD tensor vanish. Similar consideration can be used for *M_y_*; thus, the *D_xz_* and *D_zx_* terms are eliminated. Finally, only the two terms *D_xy_* and *D_yx_* are allowed in this intermediate structure of ZrTe_5_.

In general, the second-harmonic part of the NLH current related to the BCD can be represented by [18,41]:


(7) \begin{eqnarray*}
{\mathrm{\ }}J_a^{2\omega } = {\chi }_{abc} E_b^\omega E_c^\omega ,
\end{eqnarray*}

where ${\chi }_{abc}$ is the non-linear tensor and *E* is the external electric field. In the time-reversal symmetry preserved system:


(8) \begin{eqnarray*}
{\mathrm{\ }}{\chi }_{abc} = {\epsilon }^{acd} {D}_{bd}\frac{{{e}^3\tau }}{{2{\hbar }^2\left( {1 + i\omega \tau } \right)}},
\end{eqnarray*}

where $\tau $ is the relaxation time and $\epsilon $ is the Levi–Civita symbol.

Due to the symmetry constrained on the BCD, there are only left *D_xy_* and *D_yx_* so that the only possible terms are ${\chi }_{zxx},{\chi }_{xxz},{\chi }_{zyy}\ {\mathrm{and\ }}{\chi }_{yyz}$. Thus, when the external electric field is along the *x* direction, we have $J_z^{NLHE}\sim {D}_{xy}{E}_x{E}_x$, i.e. the NLH current can be measured at the *z* direction. Based on the symmetry analysis results, the non-linear susceptibility has two non-zero independent elements *d_ij_* as shown below [36]:


\begin{eqnarray*}
{\mathrm{\ }}{\chi }^{\left( 2 \right)} = \left( {\begin{array}{@{}*{3}{c}@{}} 0&\quad 0&\quad {0\quad {\mathrm{\ }}}\\ 0&\quad 0&\quad {0\quad {\mathrm{\ }}}\\ {{d}_{31}}&\quad 0&\quad {0\quad {\mathrm{\ }}} \end{array}\begin{array}{@{}*{3}{c}@{}} 0&\quad {{d}_{15}}&\quad 0\\ 0&\quad 0&\quad 0\\ 0&\quad 0&\quad 0 \end{array}} \right){\mathrm{\ }}.
\end{eqnarray*}

The coordinates *x, y, z* is selected as the axis of the crystal, namely *a*-axis, *b*-axis and *c*-axis, respectively. For an in-plane electric field *E* = (*E_x_*, 0, *E_z_*), the non-linear current density *j^(2)^* is given as 
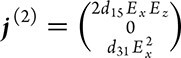
. According to Ohm's law, we have the second-order non-linear electric field 
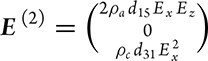
. For the applied in-plane current, 
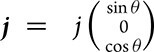
, where *j* is the current amplitude and *θ* is the angle measured from the *c*-axis. Then we can have the first-order electric field to be 
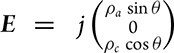
 and the longitudinal component to be ${E}_\parallel = \ j( {{\rho }_c{{\cos }}^2\theta + {\rho }_a{ {\sin }}^2\theta } )$. Moreover, the transverse component of the second-order electric field can be written as $E_ \bot ^{( 2 )} = {j}^2 {\rho }_c^3 { {\sin\, }} \theta [ { - 2{ {\cos }}^2\theta {d}_{15}{\gamma }^2 + {d}_{31}{\gamma }^2{ {\sin }}^2\theta } ]$, where γ is the anisotropy as γ *=*${\rho }_a/{\rho }_c$. Then we can have the equation below:


(3) \begin{eqnarray*}
{\mathrm{\ }}\frac{{V_ \bot ^{2\omega }}}{{{{\left( {{V}_\parallel } \right)}}^2}} = {\rho }_c\ \sin\theta \cdot \frac{{ - 2{{\cos }}^2\theta {d}_{15}{\gamma }^2 + {d}_{31}{\gamma }^2{{\sin }}^2\theta }}{{{{\left( {co{s}^2\theta + \gamma si{n}^2\theta } \right)}}^2}}.
\end{eqnarray*}

The maximal response occurs when the external electric field is along the *x-*axis and the NLH current is measured at the *z*-axis, which agrees well with our experimental results.

### First-principles calculation

Our first-principles calculations were performed using density functional theory (DFT) as implemented in the Vienna Ab initio Simulation Package (VASP) [42,43], employing the projector augmented-wave method [44]. We used the Perdew–Burke–Ernzerhof realization of the generalized gradient approximation for the exchange-correlation functional [45]. To sample the Brillouin zone, we employed a k-point mesh sampling of 17 × 5 × 5 and a plane-wave cut-off energy of 500 eV. We optimized the crystal structure with fixed lattice constants until the forces on the ions were <0.001 eV/Å. To calculate the energy difference between the centrosymmetric and non-centrosymmetric ZrTe_5_ phases, we fixed the lattice parameters for convenience. We included spin–orbit coupling due to the heavy element Te and obtained the phonon spectra using a 2 × 2 × 2 supercell with the PHONOPY package [46]. All DFT calculations were performed at 0 K.

## Supplementary Material

nwad103_Supplemental_File
