# Supplementary material for: Non-centrosymmetric topological phase probed by non-linear Hall effect
Source: Natl Sci Rev. 2023 Apr 24;11(6):nwad103. doi: 10.1093/nsr/nwad103 (PMC11081079; doi:10.1093/nsr/nwad103)
Supplement: nwad103_Supplemental_File [file nwad103_supplemental_file.docx]

Supporting information for

**Non-centrosymmetric topological phase probed by Nonlinear Hall effect**

Naizhou Wang, Jing-Yang You, Aifeng Wang, Xiaoyuan Zhou, Zhaowei Zhang, Shen Lai, Hung-Ju Tien, Tay-Rong Chang, Yuan-Ping Feng, Hsin Lin, Guoqing Chang*, Wei-bo Gao*.

*Corresponding author. Email: guoqing.chang@ntu.edu.sg, wbgao@ntu.edu.sg

**S1. The predicted structure from first-principles calculations**

The phonon spectrum of non-centrosymmetric ZrTe_5_ is presented in Figure S1. There is no imaginary-frequency mode in the whole Brillouin zone, indicating the dynamical stability of non-centrosymmetric ZrTe_5_. The lattice parameters for centrosymmetric and non-centrosymmetric ZrTe_5_ are listed in Table S1 and S2, respectively.

**S2. The band structure of centrosymmetric ZrTe_5_**

The band structure of centrosymmetric ZrTe_5_ is plotted in Figure S2. Due to the presence of both time-reversal and inversion symmetries, all energy bands are doubly degenerated. Thus, unlike the non-centrosymmetric ZrTe_5_, we did not observe the spin splitting in centrosymmetric ZrTe_5_. However, both non-centrosymmetric and centrosymmetric ZrTe_5_ are predicted to be strong topological insulators (TIs).


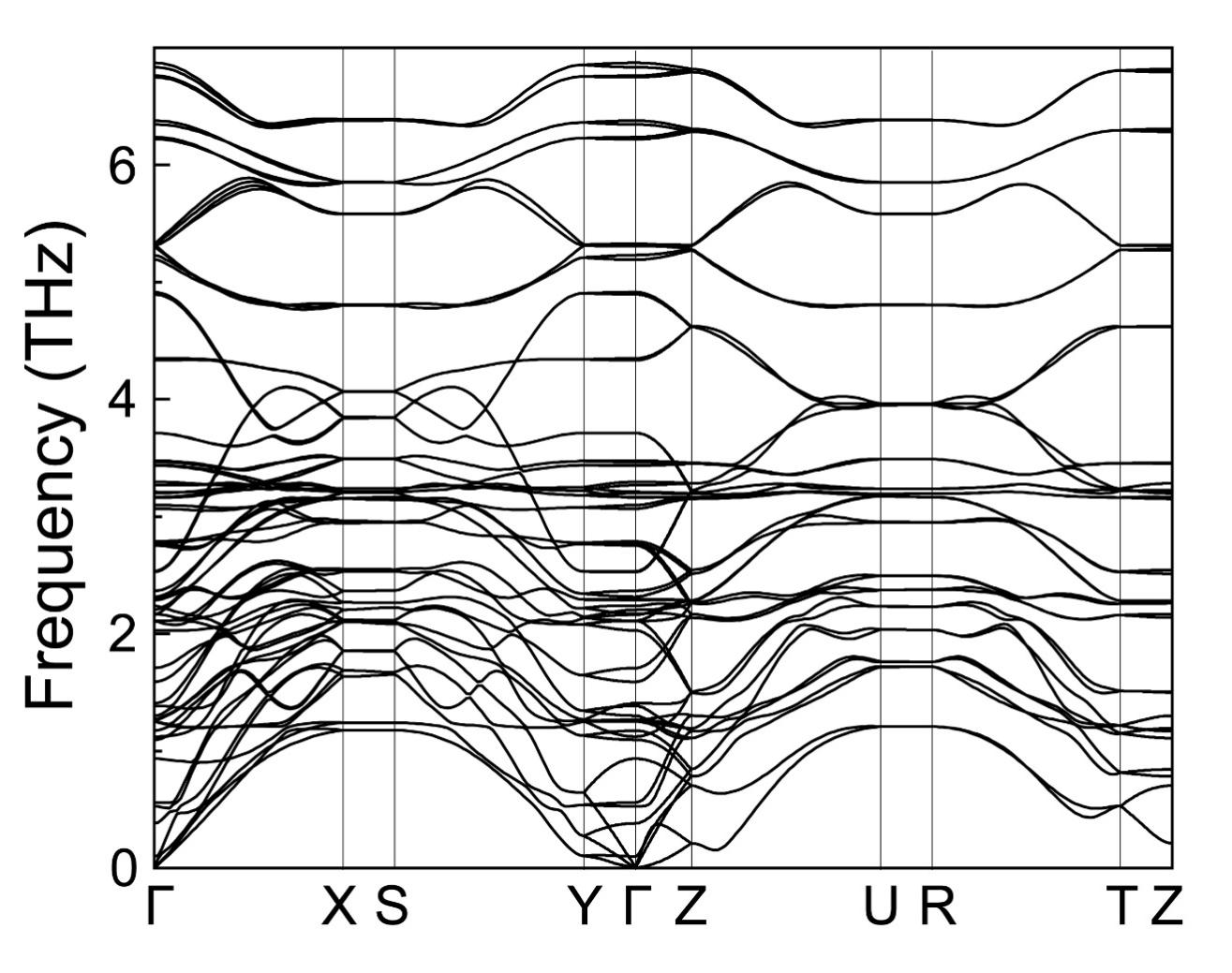


**Figure S1. The phonon spectrum of non-centrosymmetric ZrTe_5_**

| Space group | Cmcm | | | |
| --- | --- | --- | --- | --- |
| a (Å) | 3.9876 | | | |
| b (Å) | 14.502 | | | |
| c (Å) | 13.727 | | | |
| Te | 8f | 0 | 0.07010 | 0.14940 |
|  | 8f | 0 | 0.20990 | 0.56470 |
|  | 4c | 0 | 0.33650 | 0.25000 |
| Zr | 4c | 0 | 0.68570 | 0.25000 |

**Table S1.** **The lattice parameters of the centrosymmetric ZrTe_5_. The lattice parameters a, b and c are fixed at the experimental values.**

| Space group | Pna21 | | | |
| --- | --- | --- | --- | --- |
| a (Å) | 3.9876 | | | |
| b (Å) | 14.502 | | | |
| c (Å) | 13.727 | | | |
| Te | 4a | 0.00414 | 0.93223 | 0.35156 |
|  | 4a | 0.50382 | 0.83698 | 0.74987 |
|  | 4a | 0.49550 | 0.29145 | 0.93457 |
|  | 4a | 0.50449 | 0.70894 | 0.06521 |
|  | 4a | 0.49586 | 0.43247 | 0.14878 |
| Zr | 4a | 0.99589 | 0.31667 | 0.25001 |

**Table S2.** **The lattice parameters of the theoretically predicted non-centrosymmetric ZrTe_5_. The lattice parameters a, b and c are fixed at the experimental values.**


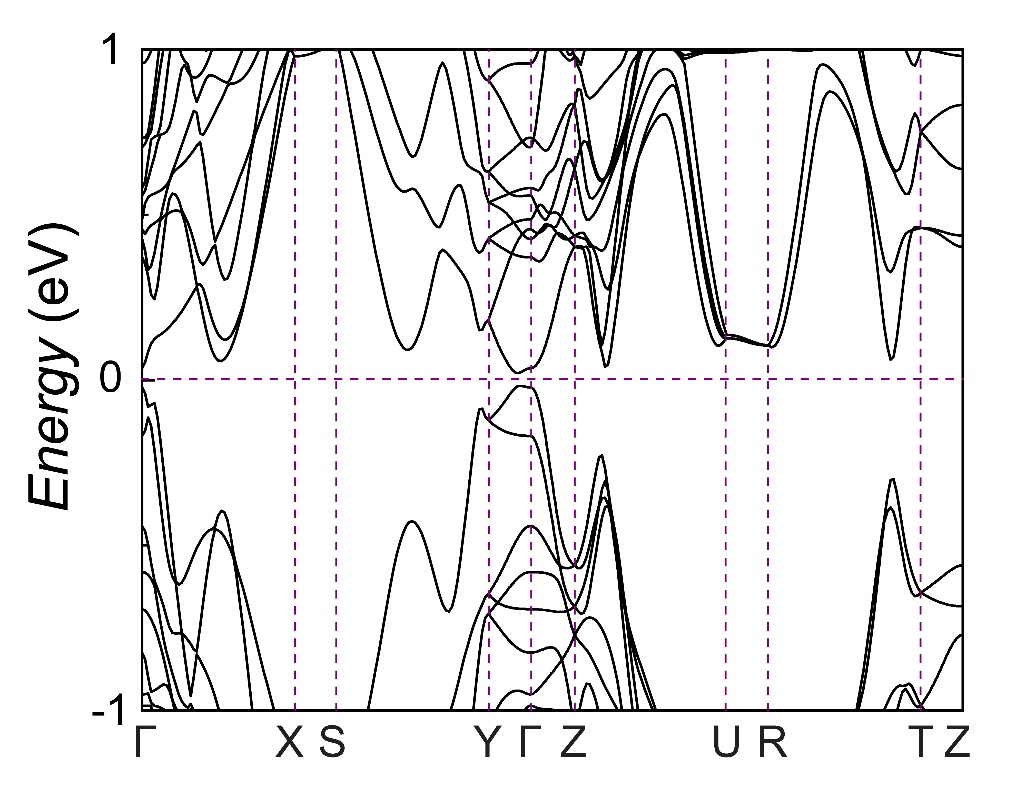


**Figure S2. The calculated band structure of centrosymmetric ZrTe_5_**

**S3. The topological properties of centrosymmetric and non-centrosymmetric ZrTe_5_**

The calculated Wilson loops show that both centrosymmetric and non-centrosymmetric ZrTe­_5_ are strong TIs, which hold the gapless Dirac cones within the bulk band gap in their surface states as shown in Figure S3.


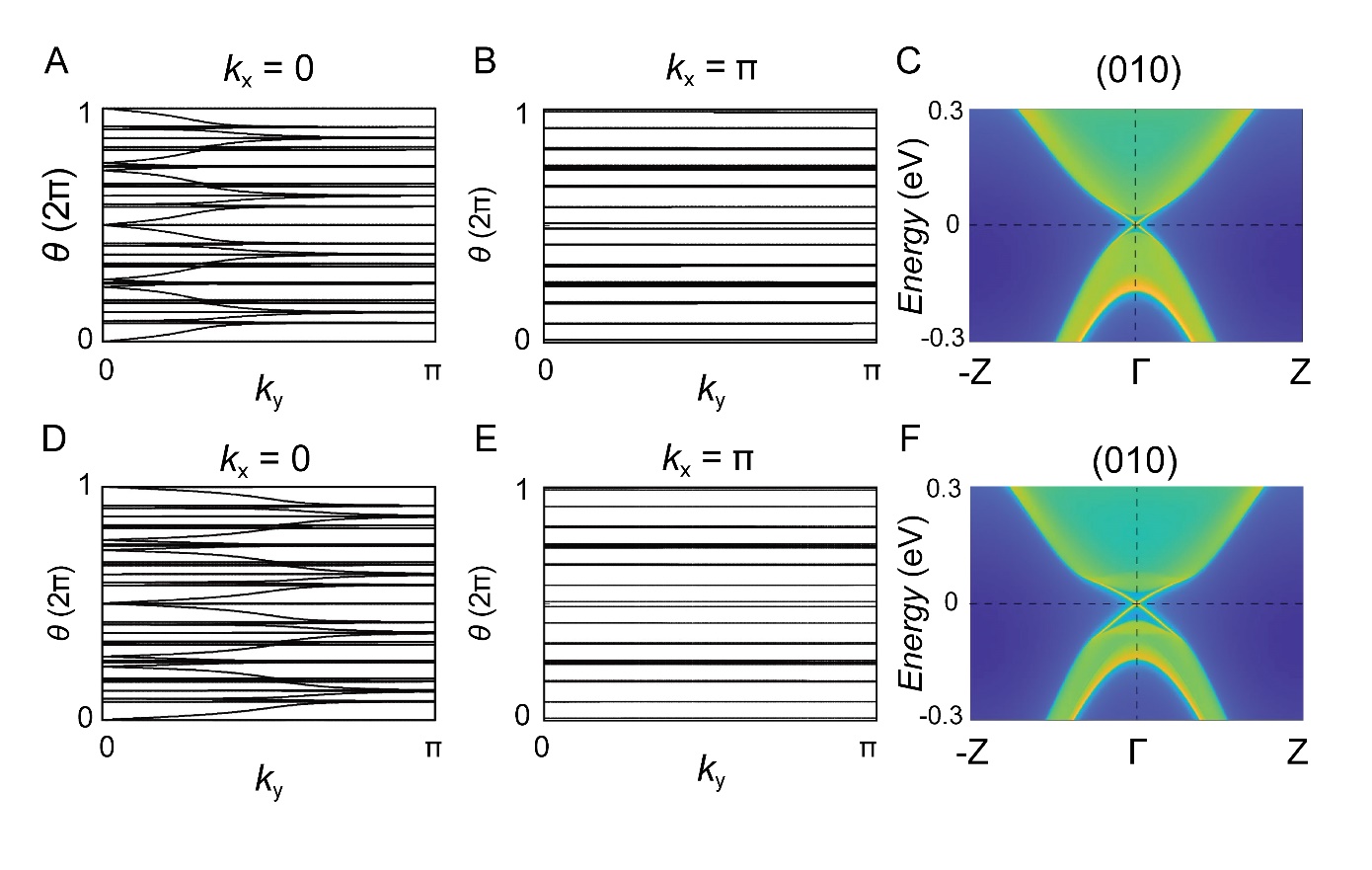


**Figure S3. The calculated topological properties of centrosymmetric and non-centrosymmetric ZrTe_5_** (**A**) and (**B**) The calculated Wilson loops *θ* (k_y_) for all occupied bands as a function of *k_y_* for the *k_x_*=0, and π planes, respectively, in centrosymmetric ZrTe_5_. (**C**) The topological surface states of centrosymmetric ZrTe_5_ in (010) plane. (**D**) and (**E**) The calculated Wilson loops θ(k_y_) for all occupied bands as a function of *k_y_* for the *k_x_*=0, and π planes, respectively, in non-centrosymmetric ZrTe_5_. (**F**) The topological surface states of non-centrosymmetric ZrTe_5_ in (010) plane.

**S4. The analysis of the peak in the temperature dependent resistivity curve for thin ZrTe_5_ flakes**

Our ZrTe_5_ device is fabricated by flux-grown samples. A resistivity peak could be observed at ~60 K in the *RT* curve as shown Figure 1(c). Here, we want to emphasize that this peak is not associated with the Lifshitz transition and is different from that observed in chemical vapor transport (CVT) grown ZrTe_5_ samples (1).

1. For the CVT-grown ZrTe_5_ sample, there exist a lot of Te vacancies and this peak is considered to be related with a temperature driven Lifshitz transition, accompanied by the change of dominate charge carrier type (1). Whereas, for our thin flake sample, the Hall coefficient shows hole-type carrier dominated and its sign remains unchanged in the whole measured temperature range (Fig S4). By using a two-carrier model, we can fit the Hall conductivity, which is shown below:

$\sigma_{xy}=\rho_{xy}/(\rho_{xx}^{2}+\rho_{xx}^{2})$, (S6)

$\sigma_{xy}=eB(\pm\frac{n_{1}\mu_{1}^{2}}{1+\mu_{1}^{2}B^{2}}\pm\frac{n_{2}\mu_{2}^{2}}{1+\mu_{2}^{2}B^{2}})$, (S7)

where *n*_1_, *n*_2_ and *µ*_1_, *µ*_2_ are the carrier density and mobility of two types of hole charge carriers, respectively, *e* and *B* is the elementary charge and applied magnetic field, respectively. The minus and plus symbols correspond to the electron and hole carriers, respectively. Figures S5(A) and S5(B) show the fitted carrier density and carrier mobility, respectively. There are two kinds of hole carriers, *h_1_* and *h_2_*. When *T*= 2 K, the hole carrier density and mobility are *n_h_*= 3.94x10^21^ m^-3^ and *µ*= 4.90 m^2^/Vs for *h_1_*, and *n_h_*=3.38x10^22^ m^-3^ and *µ*= 0.48 m^2^/Vs for *h_2_*. The carrier density is comparable to that reported in flux sample and much lower than that in the CVT samples. Moreover, with the increase of temperature, the carrier density smoothly increases and there is no sudden change. The mobility of holes with high-carrier density has little change, while the mobility of holes with low-carrier density decreases with a dip at ~80 K. Thus, according to the fitting result, there is no sudden change in the carrier density of our ZrTe_5_ devices.

2. We measured the temperature dependent resistivity curve of the bulk sample from the same batch as the exfoliated thin flake samples. There is no peak in the *RT* curve as shown in Figure S6.

Thus, according to the above analysis, the peak in the *RT* curve of thin flake ZrTe_5_ device is not associated with a Lifshitz transition. One possible reason for this peak might be the higher hole carrier density compared with the bulk sample due to the fabrication process, which has also been observed in other thin flake ZrTe_5_ samples (2, 3).


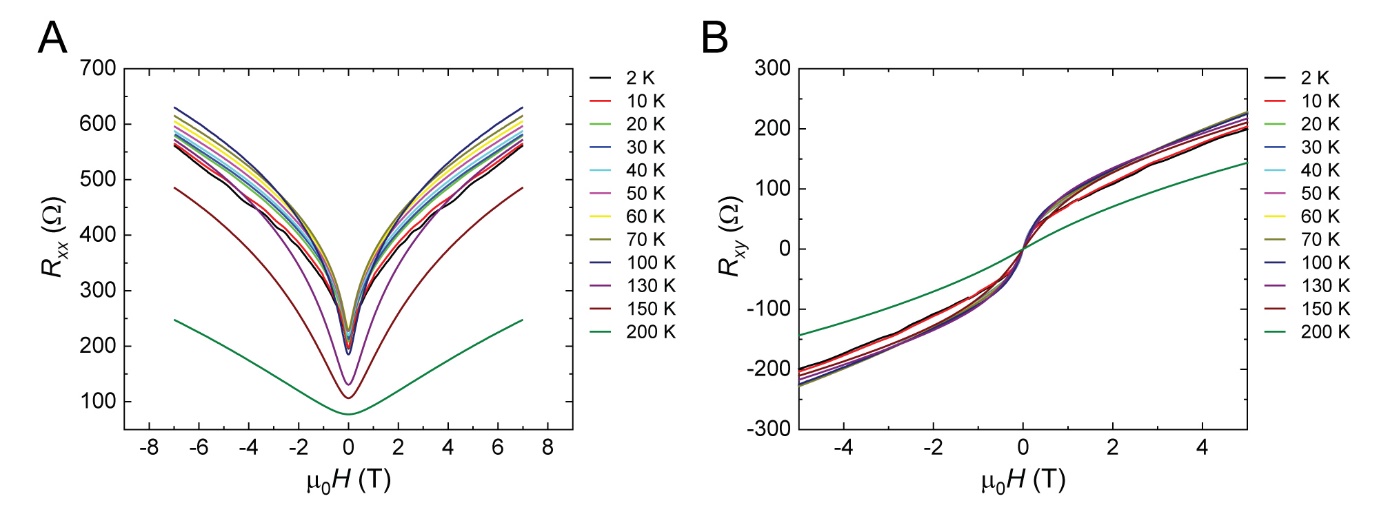


**Figure S4.** **The magnetic field dependent (A) longitudinal and (B) transverse resistance of thin flake ZrTe_5_.**


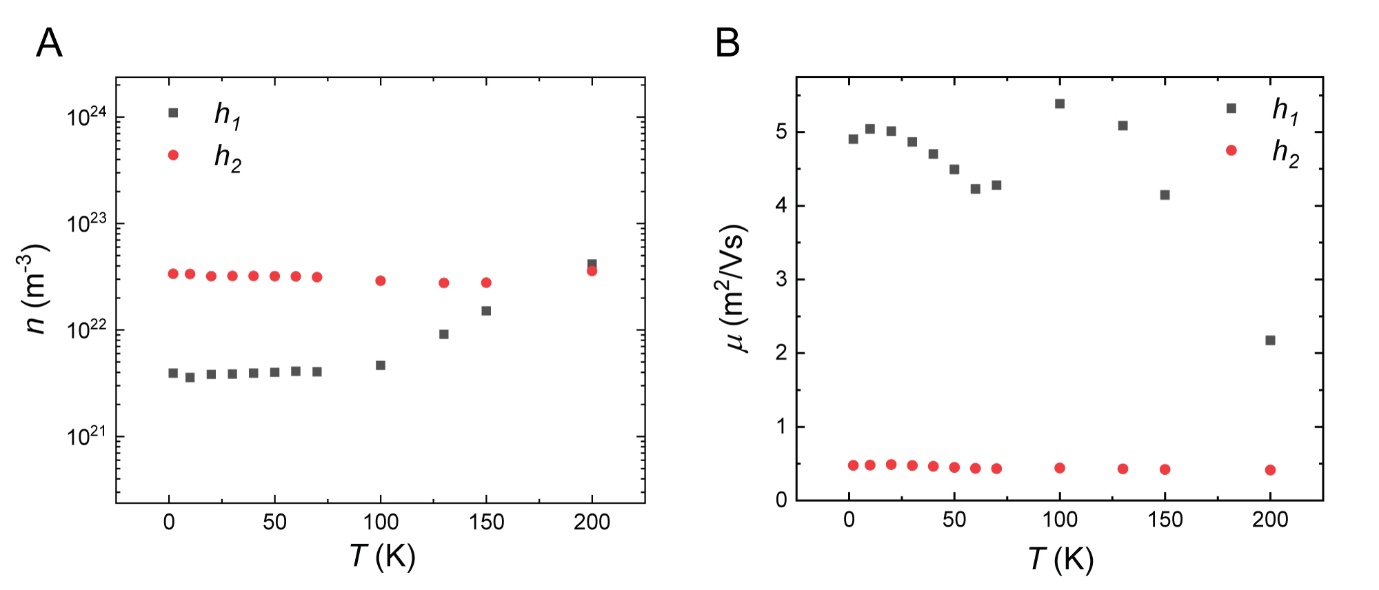
**Figure S5.** **Carrier density and mobility of thin flake ZrTe_5_ sample** (**A**) The temperature dependent carrier density of thin flake ZrTe_5_ fitted from the Hall conductivity *σ_xy_*. Two kinds of hole carriers are extracted from the fitting results. (**B**) The temperature dependent carrier mobility of thin flake ZrTe_5_ fitted from the Hall conductivity *σ_xy_*.


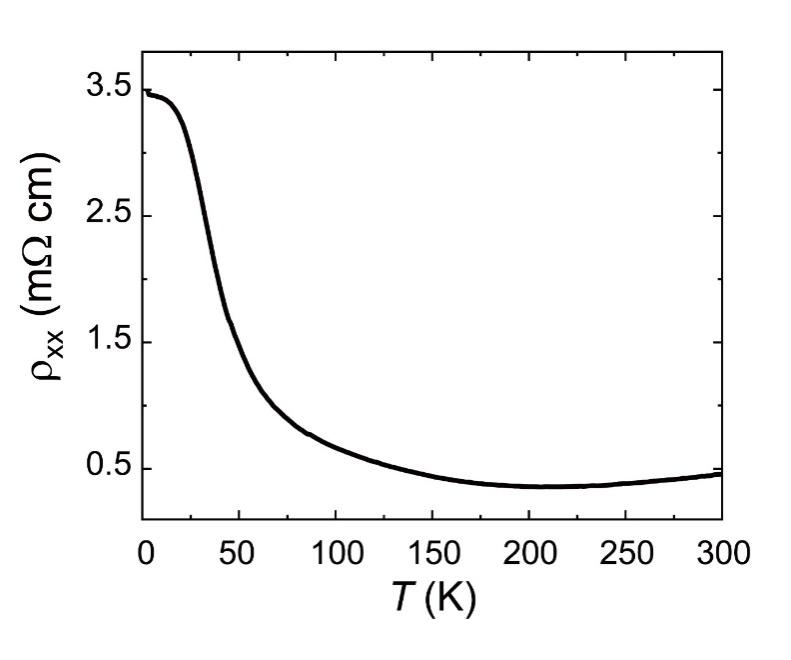


**Figure S6.** **The temperature dependent resistivity of a bulk ZrTe_5_ sample from the same batch as the exfoliated thin flake samples.**

**S5. Analysis of the Shubnikov–de Haas oscillation in ZrTe_5_**

In order to investigate the electronic structure and topological nature of the non-centrosymmetric ZrTe_5_, we carry out the Shubnikov-de Haas oscillation (SdHO) analysis in a bulk ZrTe_5_ sample. We first discuss the SdHO when the magnetic field is applied along *y*-axis. Figure S7(A) shows the magnetic field dependent resistivity at different temperatures for ZrTe_5_ sample when the applied magnetic field is parallel to the *y*-axis. A pronounced Shubnikov-de Haas (SdH)


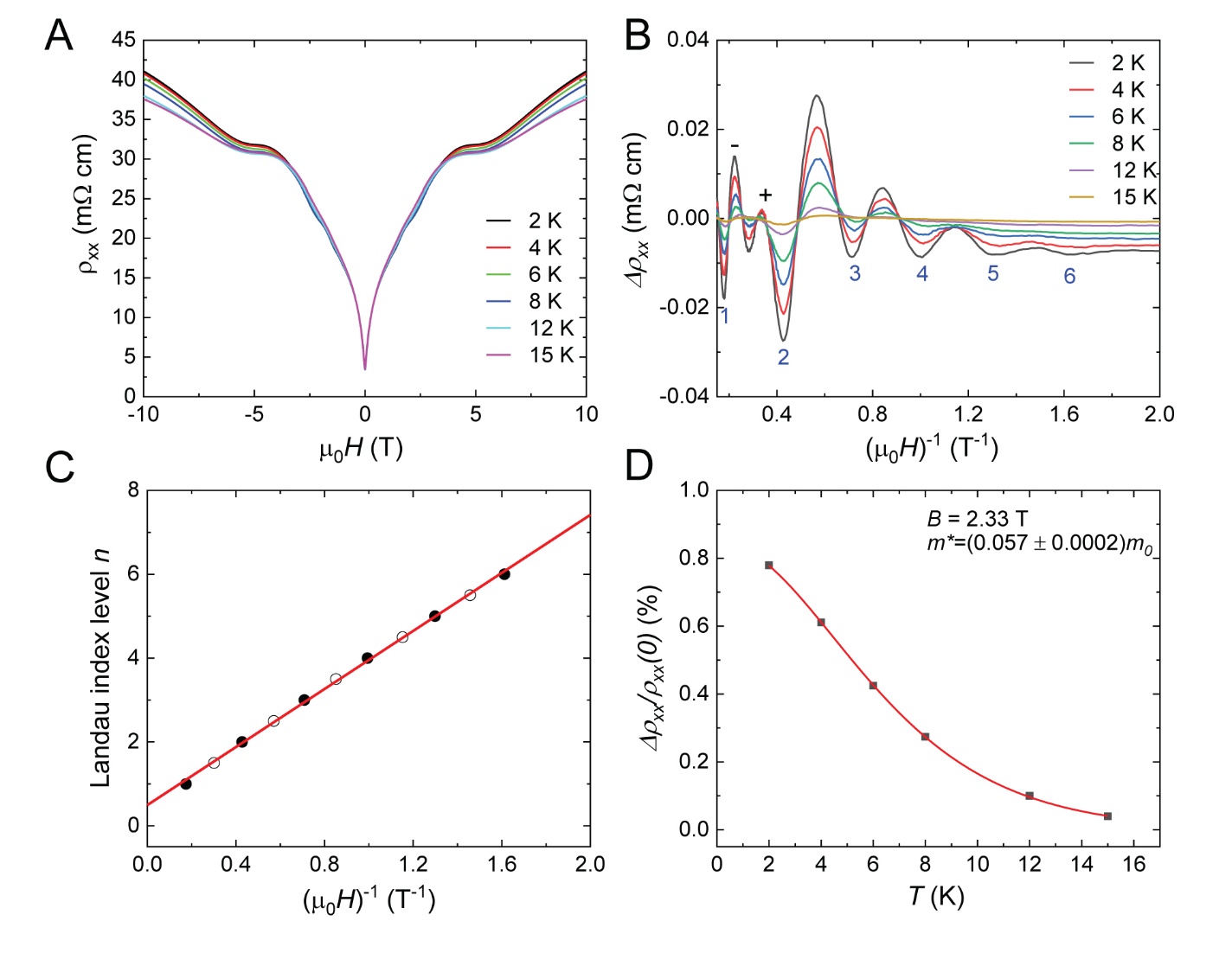


**Figure S7.** **Quantum oscillation in ZrTe_5_** (**A**). The magnetic field dependent resistivity for ZrTe_5_ at varied temperatures. The applied magnetic field is along the *y*-axis and the current along *x*-axis. (**B**). Δ*ρ*_xx_ as a function of inverse magnetic field at different temperatures. The integer Landau indices are labeled at the valleys of the oscillation patterns. Between the *n*= 1 and *n* = 2 Landau levels, a prominent Zeeman splitting could be observed. (**C**). The Landau index *n* as a function of inverse magnetic field 1/μ_0_*H*. (**D**). The oscillation amplitude as a function of temperature at 2.33 T. Red line represents the fitting result of amplitude temperature factor.

oscillation could be observed. By subtracting a smooth background, the oscillation patterns are obtained as shown in Figure S7(B). The quantum oscillation of resistivity could be described by the Lifshitz-Kosevich (LK) formula (4, 5):

$\Delta\rho\propto R_{T}R_{D}R_{S}cos[2\pi(\frac{F}{H}+\gamma-\delta)]$, (S8)

where *F* is the quantum oscillation frequency, *R_T_*, *R_D_*, and *R_s_* are the amplitude factors related to the temperature, scattering and spin splitting, respectively, *γ* is the Onsager phase factor and *δ* is the phase shift factor depending on the Fermi surface dimension. The SdHO can only be observed in low magnetic field and the system enters quantum limit at ~ 5 T with *B* // *y*-axis, suggesting a small Fermi surface in it. In our ZrTe_5_ samples, the $\rho_{xx}/\rho_{xy}$ is about 2-8 in the oscillation, then


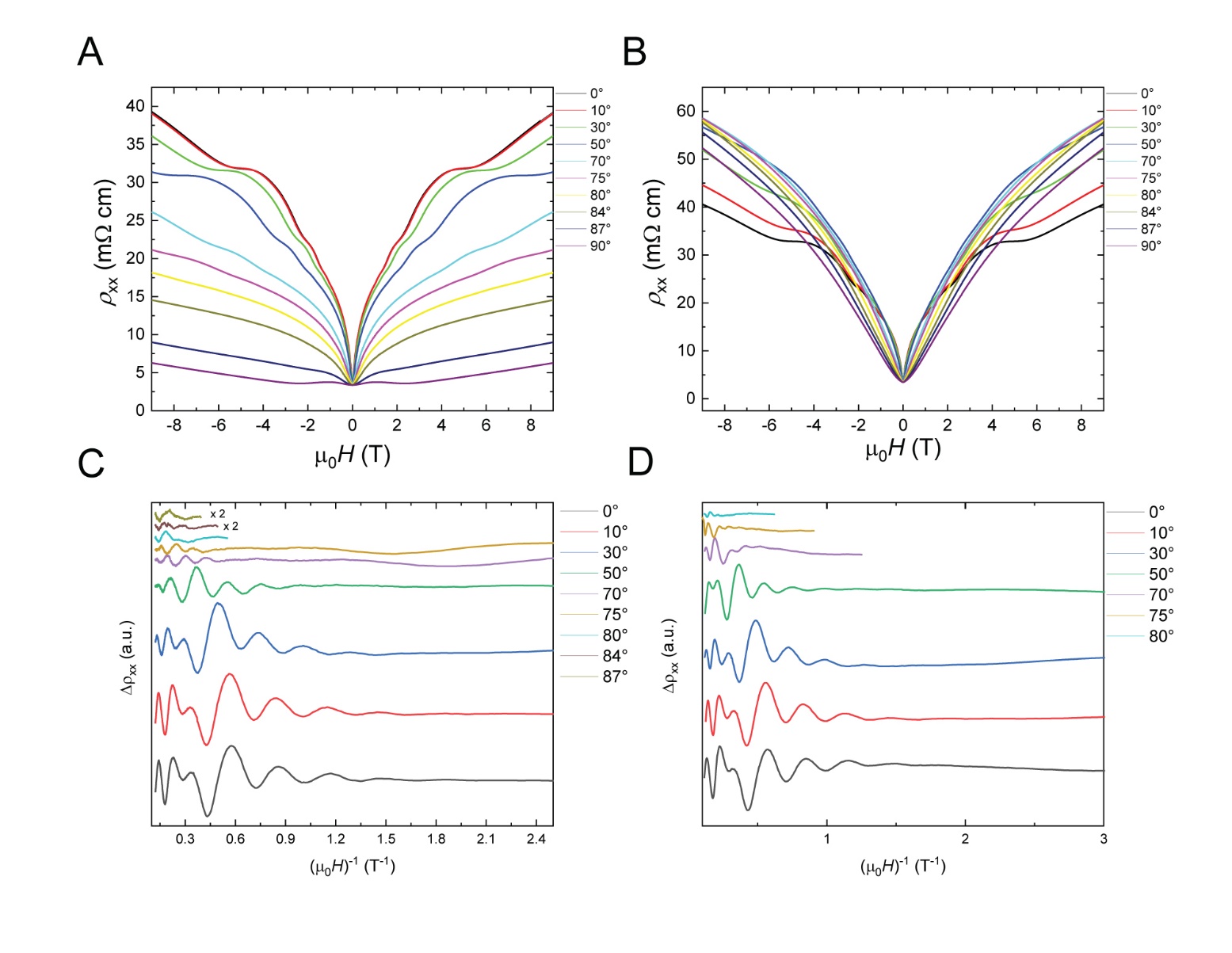


**Figure S8.** **The magnetic field dependent resistivity with rotated magnetic field in different planes.** (**A**) and (**B**) The magnetic field dependent resistivity for ZrTe_5_ with rotated magnetic field in the *yz* and *xy* planes, respectively. (**C**) and (**D**) The oscillation component Δ*ρ*_xx_ as a function of inverse magnetic field with rotated magnetic field in the *yz* and *xy* planes, respectively.

we have $\sigma_{xx}=\frac{\rho_{xx}}{\rho_{xx}^{2}+\rho_{xy}^{2}}$ ~ $\frac{1}{\rho_{xx}}$. Thus, the resistivity is totally out of phase with conductivity, and we choose the valley in the resistivity curve as the integer Landau indices. By plotting the Landau index *n* versus 1/μ_0_*H* (as shown in Figure S7(C)), we can acquire the oscillation frequency to be 3.55 T which yields a tiny cross-sectional area *S_F_* = 0.0339 nm^-2^. This finding is consistent with previous report and suggests a quite small Fermi pocket. The extracted intercept of Landau index *n* versus 1/μ_0_*H* gives a value of –*γ*+*δ* = 0.46±0.05. According to the relationship that *δ*=1/2-*Φ_B_*/2π where *Φ_B_* is the Berry phase, our SdHO analysis suggests a parabolic band dispersion with 2π when the magnetic field is along *y*-axis, which is consistent with recent reports (6, 7). In contrast, for a massless Dirac electron system with linear dispersion, the Berry phase equals to π (8). By fitting the SdHO at different temperatures, we get a tiny cyclotron mass *m^*^*= (0.0570±0.0002)*m_e_* as shown in Figure S7(D). Another feature extracted from the SdHO with the magnetic field along *y*-axis is the prominent spin splitting between *n* = 2 and *n* = 1 Landau levels. By extracting the spin-splitting


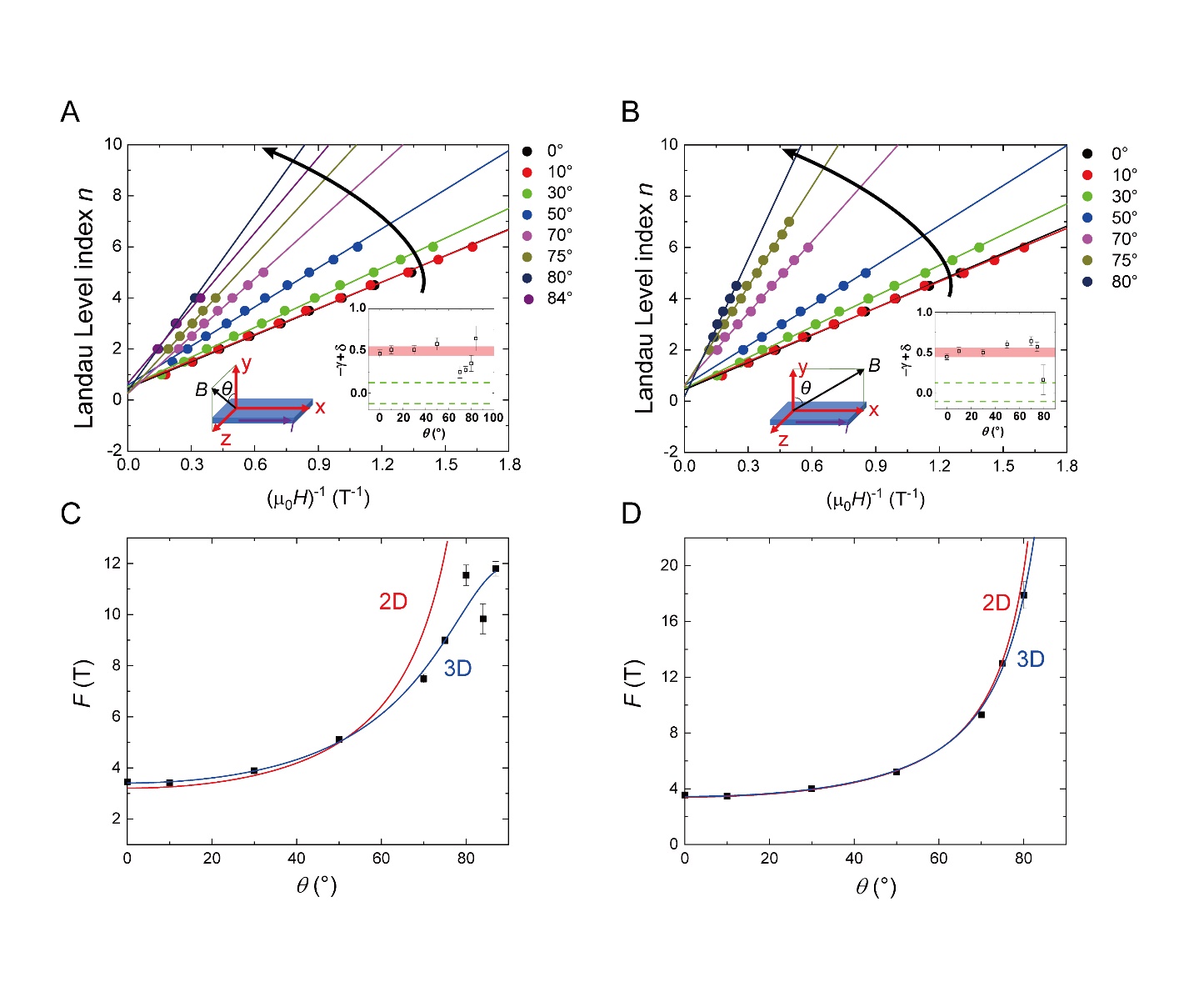


**Figure S9.** **Landau index and oscillation frequency with rotated magnetic field in different planes.** (**A**) The Landau index *n* as a function of inverse magnetic field 1/μ_0_*H* with magnetic field rotating in the *yz* plane. The angle *θ* is defined as the magnetic field direction deviated from *y*-axis. Inset shows the Onsager phase factor versus tilt angle *θ*. (**B**) The Landau index *n* as a function of inverse magnetic field 1/μ_0_*H* with magnetic field rotating in the *xy* plane. The angle *θ* is defined as the magnetic field direction deviated from *y*-axis. Inset shows the Onsager phase factor versus tilt angle *θ*. (**C**) and (**D**) The oscillation frequency *F* as a function of tilt angle *θ* for the rotating magnetic field in the *yz* and *xy* planes, respectively. Red curves represent the fitting of the 2D model, while the blue curves represent the fitting of a 3D ellipsoidal Fermi surface.

parameter $S=\frac{1}{2}g\frac{m^{*}}{m_{e}}$ , we can estimate the Landé*g* factor with a quite large value of 15.24 in our sample, which indicates a strong spin-orbit coupling (SOC) in ZrTe_5_ (9, 10).

Figures S8(A) and S8(B) show the magnetic field dependent resistivity for ZrTe_5_ sample with the magnetic field rotating in the *yz* and *xy* planes, respectively. By subtracting a smooth background, the oscillation patterns are obtained as shown in Figure S8(C) and S8(D).

When rotating the magnetic field in *yz* and *xz* planes, we can only observe a single dominant frequency, in accordance with previous report (11). Figures S9(A) and S9(B) show the obtained SdHO frequency *F* versus the tilt angle *θ* in the *yz* and *xy* planes, respectively. Two models are adopted to fit the experimental data, one is for a 2D Fermi surface with $F^{2D}=F_{z}/cos\theta$, and the other is for a 3D ellipsoidal Fermi surface with $F^{3D}=\frac{F_{z}\cdot F_{i}}{\sqrt{\left( F_{z}sin\theta\right)^{2}+\left( F_{i}sin\theta\right)^{2}}}$, where *i* =(*x,z*) and *z*=*y* for the crystal axis. The fitting of 3D model matches better with our experimental data, suggesting a 3D Fermi surface in our sample. Moreover, the extracted intercept of Landau index *n* versus 1/μ_0_*H* gives a value of about 0.5 for the rotating magnetic field in both the *yz* and *xy* planes, which suggests the parabolic band dispersion in both directions.

Finally, we can extract the cross-sectional area and the Fermi wavevector from the SdHO as listed in Table S4. According to these results, we can estimate the Fermi energy of our sample, and use the Fermi-energy to calculate the Berry curvature in the main text.

| **Crystal direction** | **Frequency (T)** | **Cross sectional Area(10^-4^ Å^-2^)** | ***k*_F_ (10^-3^ Å)** |
| --- | --- | --- | --- |
| *x* | 17.90±0.97 | 17.10±0.93 | 8.43±0.62 |
| *z* | 11.79±0.29 | 11.26±0.28 | 12.8±2.06 |
| *y* | 3.55±0.04 | 3.39±0.04 | 42.53±14.40 |

**Table S4. The fitted cross sectional area and Fermi wavevector from the SdHO.**

**S6. The reproducibility of non-linear Hall effect in ZrTe_5_**

We reproduce the non-linear Hall effect in two kinds of device configurations, namely the Hall bar and the circular disc devices.

1. Results from a Hall bar device B1

A thin flake ZrTe_5_ sample with Hall bar configuration is fabricated. Figure S10(A) shows the optical image of device B1. The thickness of this device is ~25 nm. Figure S10(B) shows the temperature dependent resistivity of device B1. A resistivity peak could be observed at ~75 K. Figures S10(C) and S10(D) show the magnetic field dependent longitudinal and transverse resistance, respectively. As we described before, the dominant charge carrier type is hole-like and there is no sudden change for this carrier type in the temperature range of 2-140 K. Figure S10(E) shows the longitudinal voltage versus applied current. The *I-V* curve shows good linearity, indicating the ohms contact for the sample. Figure S10(F) shows the second-harmonic Hall voltage as the function of the square of first-harmonic longitudinal voltage *V*_∥_ at different temperatures. The applied current is along the *x*-axis. The second-harmonic Hall voltage vanishes when the temperature is higher than 30 K, in accordance with the results in our main text.

**_
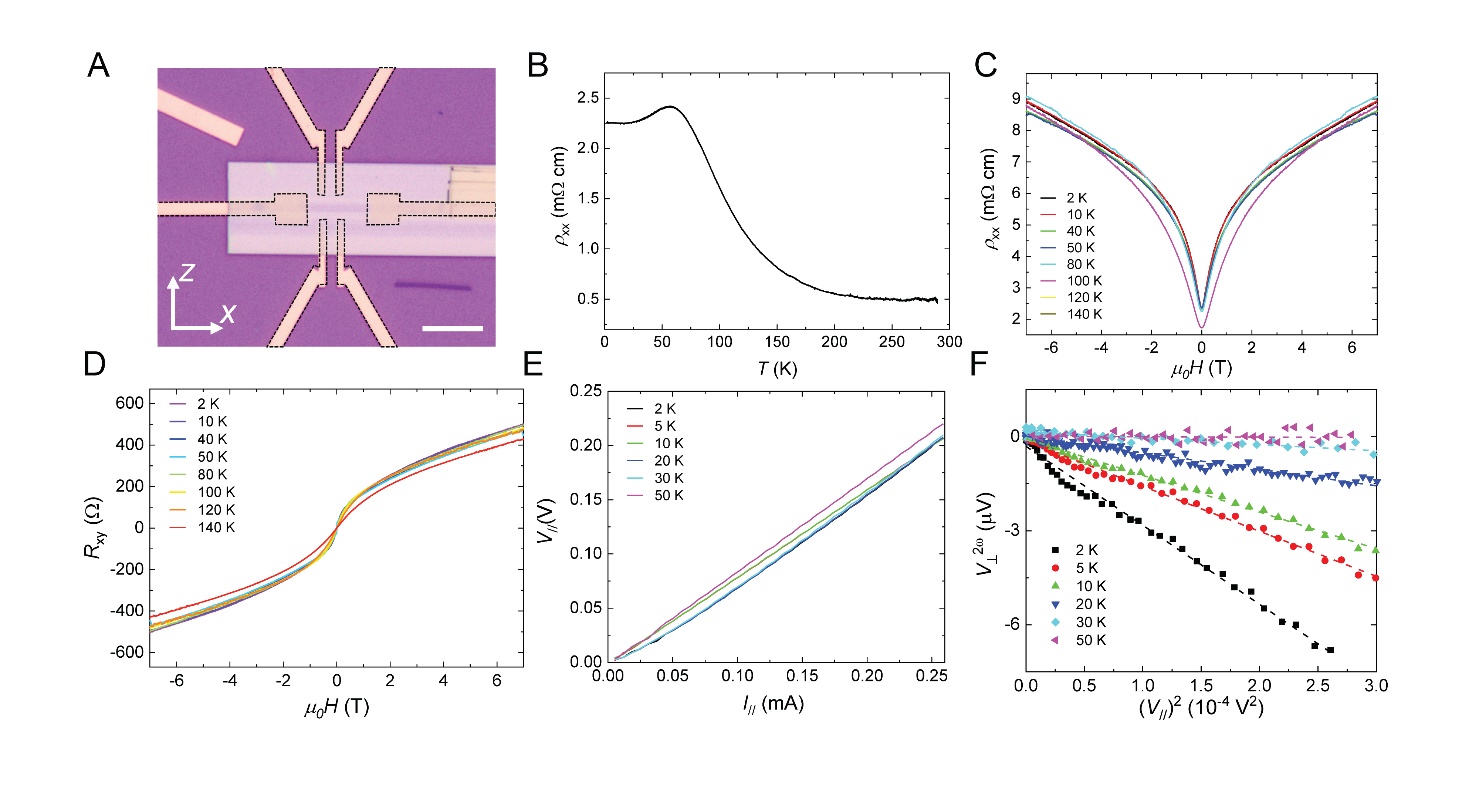
_**

**Figure S10. The non-linear Hall effect in a Hall bar device B1.** (**A**) The optical image of device B1. The scale bar is 10 μm. The thickness is about 25 nm. (**B**) The temperature dependent resistivity of device B1. A resistivity peak could be observed at ~75 K. (**C**) The magnetic field dependent longitudinal resistance of device B1 at different temperatures. The current is applied along the *x*-axis. (**D**) The magnetic field dependent transverse resistance of device B1 at different temperatures. (**E**) The longitudinal voltage as the function of applied current at different temperatures. All the curves show good linearity. (**F**) The second-harmonic Hall voltage as the function of the square of first-harmonic longitudinal voltage *V*_∥_ at different temperatures. The solid symbols are the experimental data, and the dashed lines are the linear fitting of them. The second-harmonic Hall voltage vanishes when the temperature is higher than 30 K.

2. Result from another circular disc device C1

We fabricated another circular disk device to confirm the centrosymmetric to non-centrosymmetric phase transition. Figure S11(A) shows the optical image of device C1. The thickness of this device is ~106 nm. Figure S11(B) shows the temperature dependent resistivity of device C1. A resistivity peak could be observed at ~105 K. Figure S11(C) shows the magnetic field dependent transverse resistance at different temperatures. The dominant charge carrier type is hole-like and there is no sudden change for the carrier type in the temperature range of 2-240 K. Figure S11(D) shows the longitudinal resistance as the function of injected current angles *θ*, *R*_//_ (=*V*_//_/*I*_//_). The angle dependent longitudinal resistance (*R*_//_(*θ*)) shows a quadratic angular dependence with resistance anisotropy coefficient *r* (*r*= *R_a_*/*R_c_*) of ~0.41. Figure S11(E) shows the second-harmonic Hall voltage as the function angle *θ* at T=2 K. We can also use the equation $\frac{V_{\perp}^{2\omega}}{{(V_{\parallel})}^{2}}\propto\rho_{c}sin\theta\cdot\frac{-2{cos}^{2} \theta d_{15}\gamma^{2}+d_{31}\gamma^{2}{sin}^{2} \theta}{{({cos}^{2}\theta+\gamma{sin}^{2}\theta)}^{2}}$to fit the angle dependent second-harmonic Hall voltage. Figure S11(F) shows the angle dependent second-harmonic Hall voltage at different temperatures, where the dashed lines show the fitting results. The second-harmonic Hall response gradually decreases with increasing temperature and then vanish above 30 K.


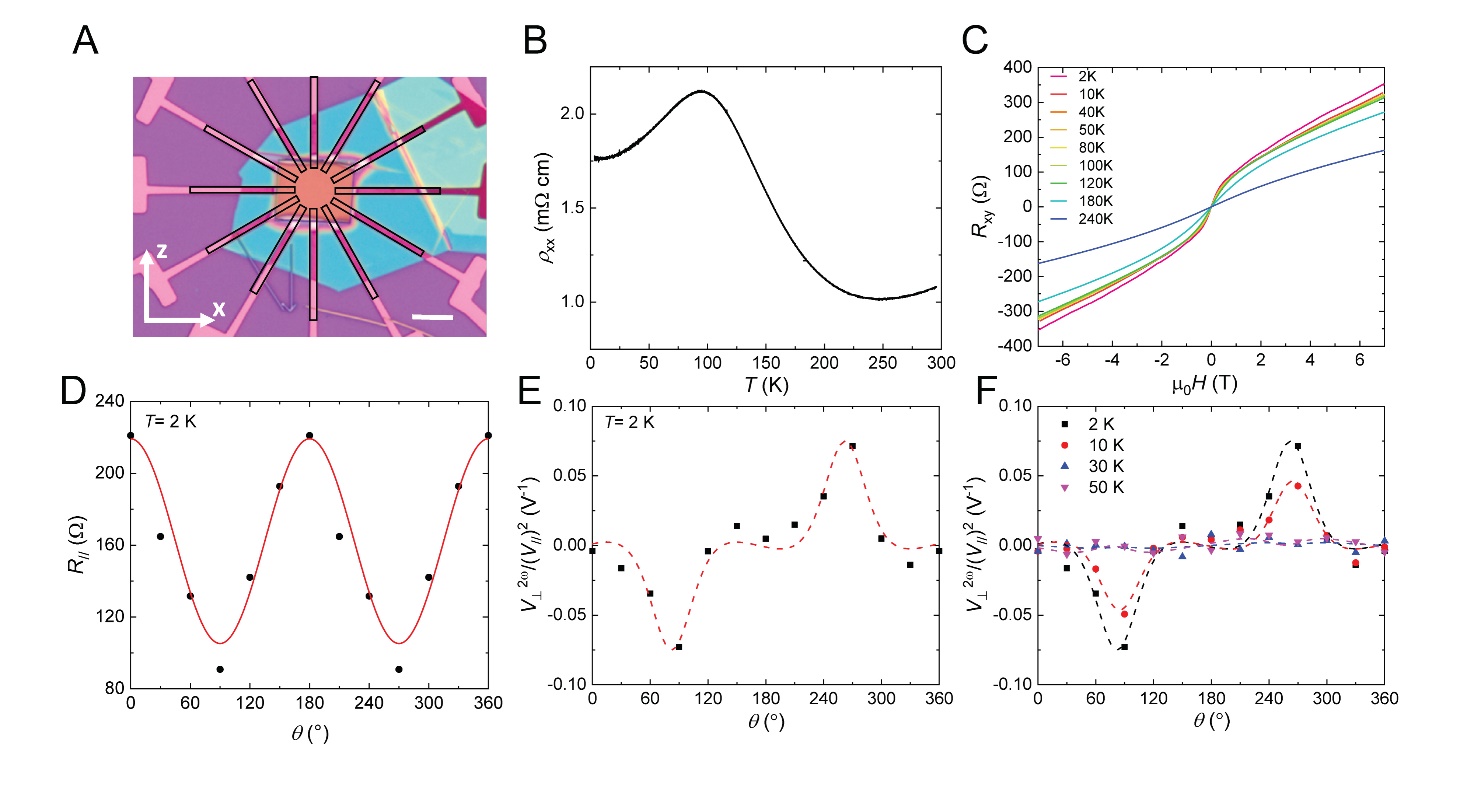


**Figure S11.** **The non-linear Hall effect in another circular disc device C1.** (**A**). The optical images of device C1. The scale bar is 20 μm. The thickness of device C1 is about 106 nm. (**B**) The temperature dependent resistivity of device C1. A resistivity peak could be observed at ~105 K. (**C**) The magnetic field dependent transverse resistance of device C1 at different temperatures. (**D**) The longitudinal resistance in different applied current directions *θ* (*θ* is the angle deviated from *z*-axis). The solid black circles represent the experimental results. The red line shows the fitting result of anisotropy resistance. (**E**) The second-harmonic Hall voltage as the function of applied current direction *θ.* The dashed line is the fitting result. (**F**) The angle dependent second harmonic Hall voltage at different temperatures. Above 30K, the second harmonic Hall response disappears.

**S7. Other possible reasons for non-linear Hall effect in ZrTe_5_**

It is possible that some extrinsic effects can also induce non-linear Hall behavior in our system. Here we give an analysis of other possible reasons and provide justification to exclude them.

7.1. The spurious capacitive coupling effect.

To exclude this effect, we measured the second-harmonic transverse as the function of injected current at different frequencies as shown in Figure S12. No obvious frequency dependence is observed in the measured frequency range of 7.777 to 37.777 Hz, which is inconsistent with the spurious capacitive coupling effect.

7.2. Contact junctions

If the contact between metal electrode and thin flake sample is not ohms like, a diode at the interface of electrode/sample may introduce a rectification effect. <1>Since this extrinsic effect strongly depends on the contact instead of the sample itself, we can use the combination of different electrodes to verify it. As shown in Figure 2(D) and Figure S11(F), a clear angle dependence of $V_{\perp}^{2\omega}$/ (*V*_//_)^2^ could be observed, which fits well with the results symmetry analysis. <2>The 2-probe DC-measurement shows good linearity as shown in Figure S13.

Thus, based on the above two evidence, we can exclude any contact junction effect.

7.3. Flake shape

Under the asymmetric sample shape, the charge carriers can move directionally by scattering the asymmetric boundaries, resulting in a high order *V*^2ω^ signal (12).

First, our exfoliated ZrTe_5_ sample is basically rectangular, which is symmetric to the principal axis, preventing such directional carrier movement. Moreover, this extrinsic signal can be excluded by this observation that an obvious angle dependence of $V_{\perp}^{2\omega}$/ (*V*_//_)^2^ could be observed, which fits well with the results of symmetry analysis.

7.4. Thermoelectric effect

The applied current may also produce the Joule heating effect on the sample. The Joule heating produces a temperature gradient and induces a thermoelectric voltage. This thermoelectric voltage follows *V*_thermoelectric_ ∝ ∆*T* ∝ *I*^2^*R* and can give rise to a second order effect (13). It should be noted that the Joule heating itself cannot show any symmetry dependence and needs to be coupled to other asymmetries, such as contact junction or flake shape. Thus, we can exclude this thermoelectric effect by our angle dependent experiment, since the angle relationship of $V_{\perp}^{2\omega}$/ (*V*_//_)^2^ can well fit with the symmetry analysis results.


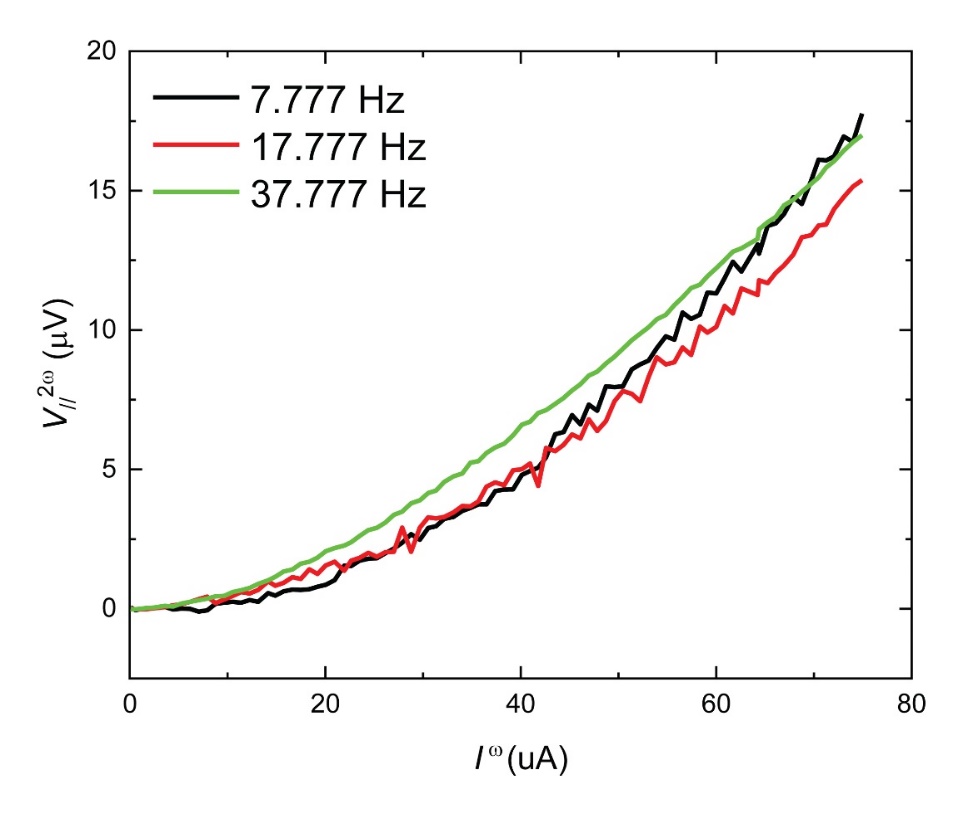


**Figure S12. The current dependent second harmonic Hall voltage under different excitation frequencies.** No obvious frequency dependence is observed.

_
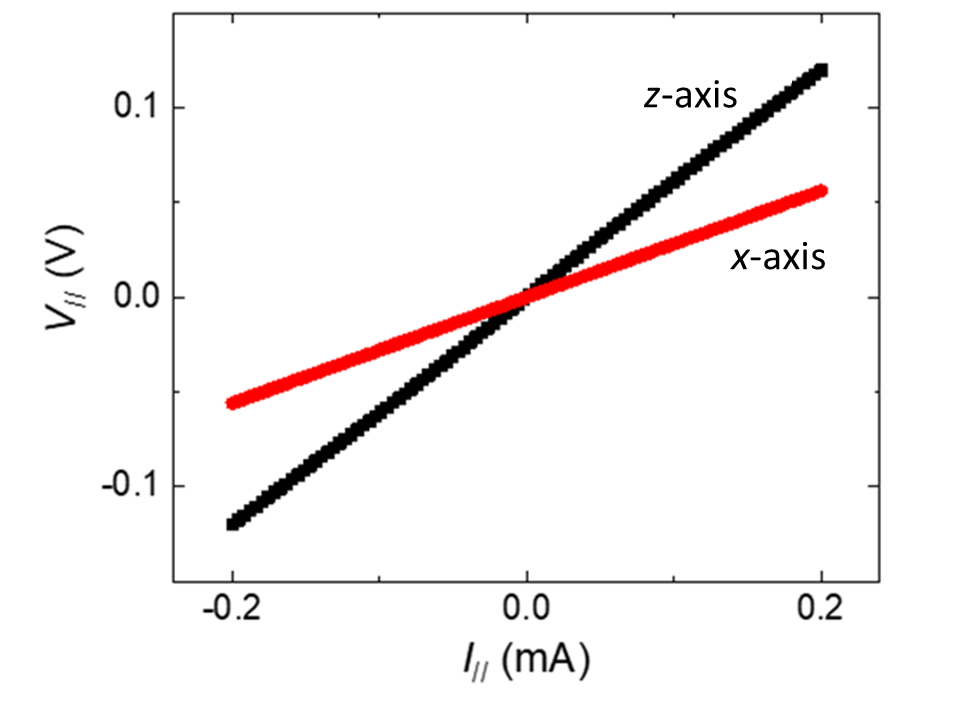
_

**Figure S13: The 2-probe DC *I-V* curve for ZrTe_5_ device.**

**S8. The current dependence of the first and second harmonic voltages of bulk ZrTe_5_ under magnetic field**

Figure S14 shows the current dependent first and second harmonic voltages of bulk ZrTe_5_ at the magnetic field of +0.05 and -0.05 T and a temperature of 2 K. The second-harmonic voltage *V*_xx_^2ω^ shows quadratic dependence on the applied current *I^ω^* with the applied current along the *x*-axis and magnetic *B*⊥*I*^ω^. Moreover, the second-harmonic voltage *V*_xx_^2ω^ is antisymmetric with applied magnetic field B, which is in accordance with the vector-product type,$V^{2\omega}=R_{0}I(\boldsymbol{P}\times\boldsymbol{B})$. However, the first-harmonic voltage *V*_xx_^2ω^ shows linear dependence on the current *I^ω^* and is symmetric to the applied magnetic field.

**_
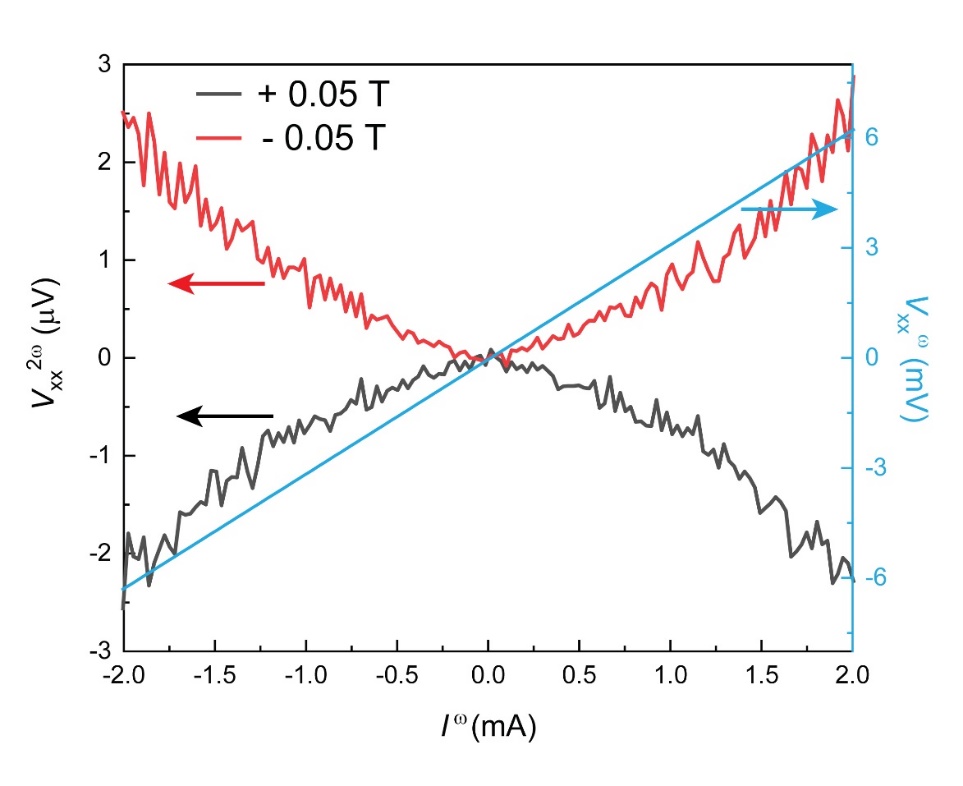
_**

**Figure S14: The current dependent first and second harmonic voltages for bulk ZrTe_5_ under the magnetic field of +0.05 and -0.05 T and a temperature of 2 K.**

**S9. The Berry curvature dipole dependence on Fermi level**

Figure S15 shows the Berry curvature dipole dependence on the Fermi level. The nonlinear Hall effect is proportional to the Berry curvature dipole and should have the same Fermi level dependence.

**
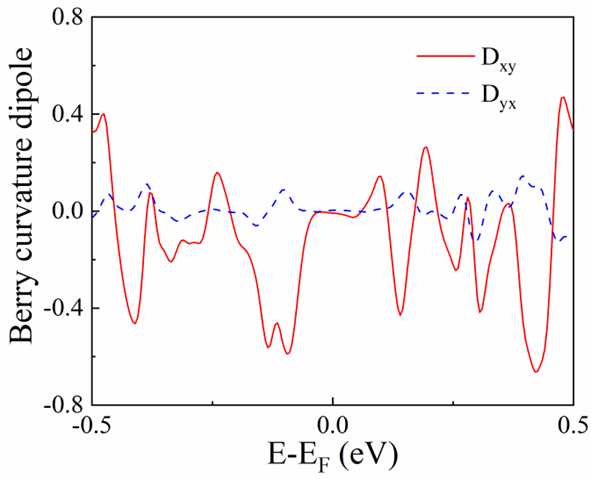
**

**Figure S15. Berry curvature dipole as a function of Fermi level.**

**S10. The magnetic field steering measurement of ZrTe_5_**

As shown in the figure S16, when both the magnetic and current are parallel to the *x*-axis of ZrTe5, namely B//J//*x*, a moderately large negative longitudinal magnetoresistance (LMR) could be observed. When the magnetic field is slightly tiled from the current direction exceeding 0.5°, the negative LMR vanished.

**
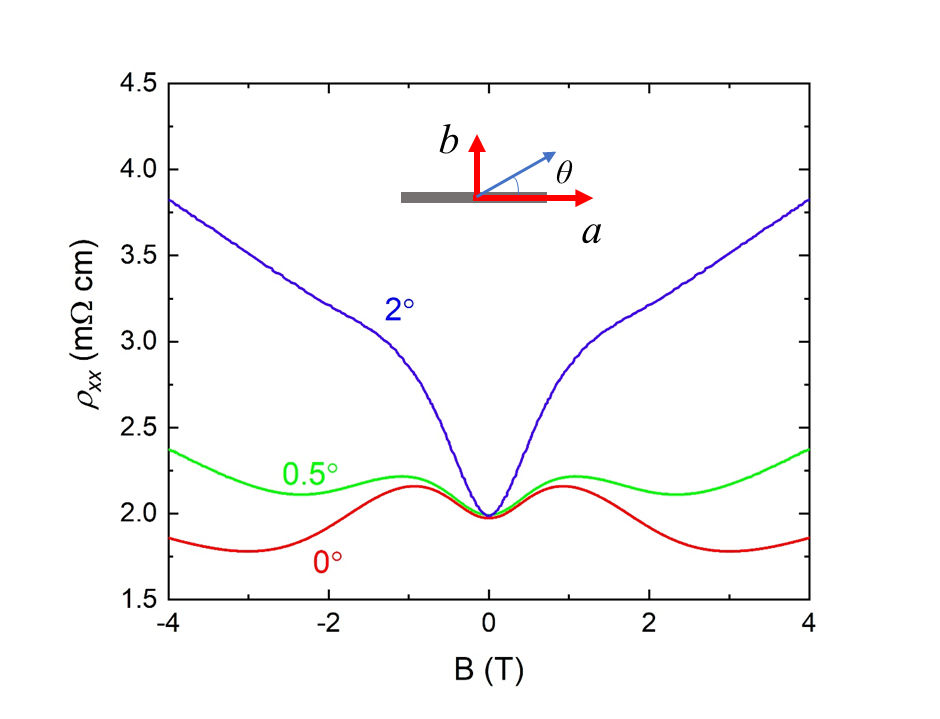
**

**Figure S16. The magnetic field dependent longitudinal resistivity of ZrTe_5_ under different angle *θ*.** The *θ* is defined as the angle deviated from the in-plane direction.

References

1. Shahi, P, Singh, DJ, Sun, JP*, et al.* Bipolar Conduction as the Possible Origin of the Electronic Transition in Pentatellurides: Metallic vs Semiconducting Behavior. *Physical Review X*. 2018; **8**(2).

2. Ge, J, Ma, D, Liu, Y*, et al.* Unconventional Hall effect induced by Berry curvature. *Natl Sci Rev*. 2020; **7**(12): 1879-85.

3. Niu, J, Wang, J, He, Z*, et al.* Electrical transport in nanothick ZrTe_5_ sheets: From three to two dimensions. *Physical Review B*. 2017; **95**(3).

4. Shoenberg, D. *Magnetic oscillations in metals*: Cambridge university press; 2009.

5. Murakawa, H, Bahramy, MS, Tokunaga, M*, et al.* Detection of Berry's phase in a Bulk Rashba semiconductor. *Science*. 2013; **342**(6165): 1490-3.

6. Liang, T, Lin, J, Gibson, Q*, et al.* Anomalous Hall effect in ZrTe_5_. *Nature Physics*. 2018; **14**(5): 451-5.

7. Sun, Z, Cao, Z, Cui, J*, et al.* Large Zeeman splitting induced anomalous Hall effect in ZrTe_5_. *npj Quantum Materials*. 2020; **5**(1).

8. Mikitik, GP, Sharlai, YV. Manifestation of Berry's Phase in Metal Physics. *Physical Review Letters*. 1999; **82**(10): 2147-50.

9. Liu, Y, Yuan, X, Zhang, C*, et al.* Zeeman splitting and dynamical mass generation in Dirac semimetal ZrTe_5_. *Nat Commun*. 2016; **7**(1): 12516.

10. Jiang, Y, Dun, ZL, Zhou, HD*, et al.* Landau-level spectroscopy of massive Dirac fermions in single-crystalline ZrTe_5_ thin flakes. *Physical Review B*. 2017; **96**(4).

11. Tang, F, Ren, Y, Wang, P*, et al.* Three-dimensional quantum Hall effect and metal-insulator transition in ZrTe_5_. *Nature*. 2019; **569**(7757): 537-41.

12. Auton, G, Kumar, RK, Hill, E*, et al.* Graphene Triangular Ballistic Rectifier: Fabrication and Characterisation. *Journal of Electronic Materials*. 2016; **46**(7): 3942-8.

13. Dames, C, Chen, G. 1ω,2ω, and 3ω methods for measurements of thermal properties. *Review of Scientific Instruments*. 2005; **76**(12): 124902.
